# Isolation, Identification, and Biological Characterization of *Proteus mirabilis* Isolated from Beef Cattle in China

**DOI:** 10.3390/microorganisms14081668

**Published:** 2026-07-30

**Authors:** Xueli Ge, Rongjun Gong, Jiamin Ma, Yangsini Fu, Jiahao Chen, Manting Li, Qinghong Guo, Xinchao Liu, Wenchao Li

**Affiliations:** Anhui Province Key Laboratory of Animal Nutritional Regulation and Health, College of Animal Science, Anhui Science and Technology University, Fengyang 233100, China; gexueli@ahstu.edu.cn (X.G.); yjs2024382@ahstu.edu.cn (R.G.); yjs2024394@ahstu.edu.cn (J.M.); qiqi2mdwn@163.com (Y.F.); chenjiahao4@ahstu.edu.cn (J.C.); yjs2024390@ahstu.edu.cn (M.L.); 11994@ahstu.edu.cn (Q.G.); 11293@ahstu.edu.cn (X.L.)

**Keywords:** *Proteus mirabilis*, isolation and identification, antimicrobial resistance, pathogenicity, whole-genome analysis

## Abstract

*Proteus mirabilis*, a member of the family *Enterobacteriaceae*, is widely distributed in the environment and commonly colonizes the gastrointestinal tract of animals. Although traditionally regarded as an opportunistic zoonotic pathogen, increasing evidence suggests that it may also contribute to bovine diseases. However, information regarding the antimicrobial resistance, virulence determinants, and genomic characteristics of bovine-associated *P. mirabilis* in China remains limited. In this study, a *P. mirabilis* strain, designated A3, was isolated from one of 19 diarrheic adult beef cattle sampled in Anhui Province, China, and comprehensively characterized through phenotypic, genomic, and pathogenicity analyses. Strain A3 exhibited typical swarming motility and a multidrug-resistant phenotype. PCR screening identified the resistance genes *aac(6′)-Ib* and *qnrA*, together with seven virulence genes (*pmfA*, *atfA*, *ureC*, *mrpA*, *ucaA*, *zapA*, and *atfC*) involved in adhesion and colonization. Whole-genome sequencing generated a 3,965,086 bp draft genome with a GC content of 38.37% and identified 85 resistance-associated genes and 147 virulence-associated genes, including multidrug efflux pumps and virulence factors related to adhesion, motility, iron acquisition, secretion systems, and host interaction. Comparative genomic analysis demonstrated high genomic similarity to other bovine-derived *P. mirabilis* isolates, with average nucleotide identity (ANI) values ranging from 99.06% to 99.32%, while pangenome analysis identified 5680 orthologous gene clusters, including 2959 core genes, indicating an open pangenome structure. Multiple insertion sequences, prophages, and genomic islands were identified, highlighting substantial genomic plasticity and adaptive potential. In a mouse infection model, strain A3 exhibited clear dose-dependent pathogenicity. All eight mice challenged with 2.22 × 10^9^ CFU/mL died within 12 h post-infection and developed severe pathological lesions in multiple organs, whereas only one of eight mice in the low-dose group died during the same period. Collectively, these findings indicate that bovine-derived *P. mirabilis* A3 combines multidrug resistance, genomic plasticity, and opportunistic pathogenic potential. This study expands current knowledge of bovine-associated *P. mirabilis* and provides a genomic and experimental basis for surveillance, risk assessment, and prevention strategies in cattle production systems.

## 1. Introduction

*Proteus mirabilis* (*P. mirabilis*) is a Gram-negative, facultatively anaerobic rod belonging to the family Enterobacteriaceae and the genus *Proteus*. It is an important opportunistic zoonotic pathogen [1]. The bacterium has a broad natural host range; it not only colonizes the intestinal mucosa of various animals but is also shed in feces, leading to its widespread distribution in soil, sewage and other environmental media. As an opportunistic pathogen, *P. mirabilis* can cause respiratory, urinary and digestive tract infections when host immunity is compromised. It can directly inflict severe damage on the upper urinary tract and is often associated with the formation of bladder and kidney stones [2,3]. Recent studies have shown that, in addition to causing diarrhea, food poisoning, and septicaemia in humans [4], *P. mirabilis* can infect a variety of animals, including companion animals [3], chickens [5], cynomolgus monkeys [6], South China tigers [7], and banded kraits [8]. The incidence and mortality of infections caused by this bacterium have been increasing in recent years, posing a threat to public health and drawing considerable attention from researchers in microbiology, epidemiology, and clinical medicine worldwide [9,10,11].

Within the current taxonomic framework, the genus *Proteus* comprises *Proteus mirabilis*, *Proteus vulgaris*, *Proteus penneri*, *Proteus hauseri*, and three unnamed genomospecies (4, 5, and 6). Among these members, *P. mirabilis* is considered the most clinically relevant and extensively studied species [12,13]. The bacterium lacks both a capsule and spores but possesses numerous fimbriae and peritrichous flagella, which confer strong motility. This motility not only facilitates host colonization but also contributes to biofilm formation, an important factor in bacterial persistence and pathogenicity [14,15]. In addition, *P. mirabilis* produces a variety of virulence factors, including urease, hemolysin, swarming-associated factors, and the secreted metalloprotease *ZapA*. These virulence determinants enhance bacterial colonization and pathogenicity and may also facilitate the dissemination of antimicrobial resistance genes [16]. Uropathogenic bacteria, including *Escherichia*, *Proteus*, and *Streptococcus* species, are commonly derived from the intestinal microbiota and can colonize the periurethral region before ascending into the urinary tract, leading to symptomatic or asymptomatic bacteriuria [17]. Previous studies have shown that bacterial adhesion to uroepithelial cells represents a critical step in the pathogenesis of *Proteus* infections [18,19].

In recent years, the cattle industry in China has undergone rapid expansion and progressive intensification, with a growing number of large-scale farms housing more than 10,000 animals and an increasing contribution of cattle production to the livestock sector [20]. Sustainable development of the industry is closely linked to animal health and production efficiency. Enteric bacterial pathogens not only impair growth performance and cause economic losses but may also serve as reservoirs of antimicrobial resistance and sources of zoonotic transmission.

Multidrug-resistant (MDR) *P. mirabilis* has been detected with increasing frequency in cattle populations worldwide, a trend that has attracted growing attention in recent years [13,16,21]. Accumulating evidence indicates that cattle-derived *P. mirabilis* isolates commonly exhibit MDR phenotypes [14,22,23]. Although bovine-origin *P. mirabilis* has received less attention than *Escherichia coli* or *Salmonella enterica* as a reservoir of antimicrobial resistance, available studies suggest that this pathogen may contribute to the dissemination of clinically relevant resistance genes [13,16]. In China, regional investigations have further supported this concern. *P. mirabilis* isolates recovered from diarrheic calves and adult cattle in different provinces have shown high resistance rates to β-lactams, tetracyclines, and fluoroquinolones, with a considerable proportion of isolates displaying MDR phenotypes [14,22,23]. A recent survey in Zhejiang Province isolated 101 bovine-origin *P. mirabilis* strains from slaughterhouses and farmers’ markets; resistance rates to ceftriaxone, amoxicillin, and erythromycin all exceeded 90%, and 89.09% of the isolates carried the ESBL-encoding gene *blaTEM* [23]. In Inner Mongolia, 14 *P. mirabilis* isolates obtained from diarrheic calves showed an MDR rate of 92.9%, with resistance rates to tetracycline, polymyxin B, and trimethoprim-sulfamethoxazole all exceeding 78.6% [14]. Similarly, bovine-origin isolates from Xinjiang exhibited diverse antimicrobial resistance patterns and virulence gene profiles [22].

Evidence from other countries also suggests the potential public health relevance of food-animal-associated *P. mirabilis* [13,16,24]. In Brazil, Sanches et al. [24] reported that P. mirabilis isolates from retail beef showed marked genetic similarity to human clinical isolates, suggesting that food-animal-derived strains may represent a potential route for the transmission of antimicrobial resistance genes to humans. The systematic review by Liu et al. [13] comprehensively assessed the zoonotic risks of *P. mirabilis* in animals and animal-derived foods, further highlighting the ecological importance of this bacterium in cross-species transmission. From a One Health perspective, Hasona et al. [16] noted that virulence factors and antimicrobial resistance genes may be co-disseminated between human and animal hosts. A recent global genomic analysis of 3403 *P. mirabilis* genomes showed that isolates from China carried the highest number of antimicrobial resistance genes, with β-lactamase and carbapenemase genes being particularly widespread [21].

Important knowledge gaps remain. Most available studies on bovine *P. mirabilis* are limited to regional isolation, antimicrobial susceptibility testing, or the detection of selected resistance and virulence genes [13,14,22,23]. Few studies have integrated phenotypic characterization, whole-genome analysis, assessment of mobile genetic elements, and pathogenicity evaluation within a single framework [13,21]. Information regarding the antimicrobial resistance profiles, virulence gene repertoire, genomic features, and pathogenic potential of *P. mirabilis* isolated from beef cattle in China remains fragmented [14,22,23]. This limitation restricts a comprehensive understanding of the role of bovine-derived *P. mirabilis* in the emergence and transmission of antimicrobial resistance [13,16,21].

The present study characterized a multidrug-resistant bovine-derived *P. mirabilis* isolate, designated A3, focusing on its pathogenicity, antimicrobial resistance profile, resistance- and virulence-associated genes, and whole-genome features. Comparative genomic and pangenome analyses were performed to evaluate genomic diversity, mobile genetic elements, and potential evolutionary adaptation within *P. mirabilis* populations. This study aims to improve our understanding of the pathogenic potential and antimicrobial resistance of *P. mirabilis* isolated from cattle and to support future surveillance strategies. The expected outcome is to provide strain-level phenotypic and genomic evidence that may serve as a reference for future large-scale epidemiological investigations and risk assessment of bovine-origin *P. mirabilis*.

## 2. Materials and Methods

### 2.1. Sample Source

Samples were obtained from a beef cattle farm located in Panji District, Huainan City, Anhui Province, China. The farm housed a total of 150 cattle, including 100 adults and 50 calves. The barns were of semi-open brick-and-concrete construction, with dry straw used as bedding. The average floor space per animal was approximately 4 m^2^, and ventilation was provided by a combination of natural airflow and mechanical assistance. Drinking water came from a deep well and was supplied ad libitum via water troughs. The diet consisted of a total mixed ration (TMR) comprising corn silage and wheat straw as roughage, supplemented with a concentrate mixture (corn, soybean meal, wheat bran, and a premix). The animals were fed twice daily at fixed times and had free access to feed. This study was conducted from August 2025 to February 2026. In mid-August 2025, a total of 19 rectal swabs were collected from adult beef cattle with diarrhea. All sampled diarrheic adult beef cattle had not received antibiotic treatment before rectal swab collection. After sampling, each swab was placed in a sampling tube containing Brain Heart Infusion (BHI) liquid medium and sealed. The samples were transported to the laboratory within 2 h and then incubated overnight at 37 °C in a shaker incubator.

### 2.2. Bacterial Isolation and Identification

The enriched bacterial culture was streaked onto BHI agar, MacConkey agar (MAC), xylose lysine deoxycholate (XLD) agar, and Luria–Bertani (LB) agar (Haibo Biotechnology Co., Ltd., Qingdao, China) for bacterial isolation. The plates were inverted and incubated at 37 °C for 24 h. Several dozen presumptive colonies exhibiting distinct colony morphologies were selected and purified by subculturing under the same conditions (37 °C, 24 h, inverted incubation).

Single colonies with morphological characteristics consistent with *P. mirabilis* were selected for Gram staining and microscopic examination. The selected colonies were then inoculated into test tubes containing 10 mL of BHI liquid medium and incubated overnight in a shaking incubator at 37 °C. The purified bacterial suspension was aseptically aspirated and inoculated into Enterobacteriaceae biochemical identification tubes (Microbiological Reagent Co., Ltd., Hangzhou, China) for biochemical identification. The tubes were incubated at 37 °C for 24–48 h, and the test was repeated three times. The results were interpreted according to the observed reactions, recorded, and statistically analyzed.

Based on colony morphology, biochemical characteristics, and 16S rRNA gene sequencing, only one *P. mirabilis* isolate was recovered from the 19 samples examined. This isolate was designated strain A3 and was used for subsequent antimicrobial susceptibility testing, pathogenicity assessment, and whole-genome sequencing analyses.

### 2.3. 16S rRNA Analysis of the Isolated Strain

DNA templates were extracted using the traditional boiling lysis method. Within a clean bench, a single purified colony was picked and placed into a 1.5 mL sterile EP tube containing 100 μL of double-distilled water. A 100 μL bacterial suspension was prepared by vortexing thoroughly to disperse the colonies, which was then numbered. The tube was heated in a boiling water bath for 8 min, then placed on ice for 10 min. After cooling, the tube was centrifuged at 12,000 r/min for 2 min in a refrigerated centrifuge. The supernatant was carefully aspirated and stored at −20 °C for later use. Bacterial universal primers [25] (27F: 5′-CGTGCCTAATACATGCAAGTCGAAC-3′; 1492R: 5′-ACGACTTCACCCCAATCATCTATCC-3′) were synthesized by Shanghai Sangon Biotech Co., Ltd. (Shanghai, China). PCR amplification was performed in a 50 μL reaction mixture containing 25 μL of 2 × Taq PCR MasterMix (Beijing Solarbio Science & Technology Co., Ltd., Beijing, China), 2 μL of forward primer, 2 μL of reverse primer, 5 μL of DNA template, and 16 μL of double-distilled water. The amplification conditions were as follows: initial denaturation at 95 °C for 5 min; followed by 35 cycles of denaturation at 95 °C for 30 s, annealing at 55 °C for 30 s, and extension at 72 °C for 1 min; and a final extension at 72 °C for 10 min. The PCR products were separated by 1.2% agarose gel electrophoresis and visualized using a gel imaging system. Positive PCR products meeting the expected size were sent to Sangon Biotech (Shanghai, China) Co., Ltd. for sequencing. The 16S rRNA gene sequences obtained from sequencing were submitted to the NCBI (National Center for Biotechnology Information) database. Homology analysis was performed using the BLAST (https://blast.ncbi.nlm.nih.gov, accessed on 10 September 2025) program against reference 16S rRNA sequences of standard strains available in the database, and a phylogenetic tree was constructed using MEGA software (version 11.0.13).

### 2.4. Growth Curve Determination

Activated bacterial culture was inoculated at 2% (*v*/*v*) into 10 mL of sterile BHI medium in triplicate. The cultures were incubated at 37 °C in a shaker. Samples were taken every 1 h, and the absorbance at 600 nm (OD_600_) was measured using BHI medium as a blank control. A growth curve was plotted with time on the x-axis and OD_600_ on the *y*-axis.

### 2.5. Antimicrobial Susceptibility Testing

The Kirby-Bauer disk diffusion method was used in this study [26]. The bacterial suspension was adjusted to a concentration of 1.0 × 10^8^ CFU/mL. Under aseptic conditions, 100 μL of the diluted bacterial suspension was evenly spread onto Mueller-Hinton (MH) agar plates. After allowing the liquid to absorb for 3–5 min at room temperature, six antibiotic disks were evenly placed on the surface of a single culture medium. The plates were incubated inverted at 37 °C for 18–24 h. The test was performed in triplicate. After incubation, the diameters of the inhibition zones were measured, and the average values were calculated. Susceptibility to 26 antibiotics was evaluated using commercially available antimicrobial susceptibility disks (Hangzhou Microbial Reagent Co., Ltd., Hangzhou, China) covering β-lactams (ceftriaxone, cefazolin, cefoperazone, cefuroxime, ceftazidime, cefalexin, ampicillin, piperacillin, cefradine, carbenicillin, and imipenem), aminoglycosides (amikacin, kanamycin, gentamicin, and neomycin), tetracyclines (doxycycline, minocycline, and tetracycline), quinolones (ofloxacin, ciprofloxacin, and norfloxacin), macrolides (erythromycin, midecamycin, and azithromycin), amide alcohols (chloramphenicol), and sulfonamides (trimethoprim-sulfamethoxazole) were determined according to the Clinical and Laboratory Standards Institute (CLSI) M100-ED30: 2020 guidelines [27].

### 2.6. PCR Detection of Resistance Genes and Virulence Genes

Based on the previous studies [28], seven pairs of primers for drug resistance genes and eight pairs of primers for virulence genes were synthesized (Shanghai Sangon Biotech Co., Ltd., China) and used to detect the corresponding genes in the isolated strains by PCR. The primer information and the annealing conditions for PCR amplification are listed in Table 1. Other amplification conditions and the PCR reaction system were the same as those described in Method 2.3. The PCR products were separated by electrophoresis on a 1.2% agarose gel.

### 2.7. Whole-Genome Analysis of P. mirabilis Strain A3

#### 2.7.1. Overview of Genome Data

Whole-genome sequencing of strain A3 was performed by Chuzhou General Biotechnology Co., Ltd. (Chuzhou, China). Before formal genome assembly, the basic characteristics of the genome (e.g., genome size) were estimated using reads from short-insert libraries (insert size < 1500 bp).

#### 2.7.2. Genome Assembly and Annotation

Raw sequencing reads were quality-filtered before assembly. De novo genome assembly was performed using SPAdes (v3.15.5) [29] with default settings. Scaffolds longer than 500 bp and with an average sequencing depth greater than 10× were retained for downstream analyses. The assembled genome was subsequently polished using Pilon v1.24 to correct single-nucleotide errors and small insertion/deletion variants, resulting in a high-quality final genome assembly.

#### 2.7.3. Genome Map Construction

A circular genome map was generated using Circos (v0.69-9) [30]. Genomic features, including guanine-cytosine (GC) content, GC skew, coding sequences (CDSs), non-coding RNAs (ncRNAs), gene density, genomic variations, and Clusters of Orthologous Groups (COG) functional classifications, were visualized. The 20 longest scaffolds (>5000 bp) were selected for graphical representation.

#### 2.7.4. GO Annotation Analysis

Functional annotation based on Gene Ontology (GO) terms was performed using InterProScan (v5.67-99.0). GO annotations were categorized into three major domains: biological process, molecular function, and cellular component. GO Slim annotation was subsequently conducted using Map2Slim (Gene Ontology Consortium, http://www.geneontology.org/ (accessed on 10 December 2025)).

#### 2.7.5. COG Annotation Analysis

Protein-coding genes were functionally annotated using eggNOG-mapper (v2.1.12) based on the eggNOG (v5.0) database. Orthologous group assignment and Cluster of COG functional classification were performed through sequence similarity searches. Identified genes were categorized into 25 COG functional classes.

#### 2.7.6. KEGG Annotation Analysis

Annotation of metabolic pathways was conducted using the KEGG Automatic Annotation Server (KAAS; Kyoto Encyclopedia of Genes and Genomes Automatic Annotation Server) [31] with the bidirectional best-hit (BBH) method. Predicted protein sequences were assigned to KEGG Orthology (KO) identifiers, and metabolic pathway reconstruction was performed based on the Kyoto Encyclopedia of Genes and Genomes (KEGG) database (https://www.genome.jp/kegg/ (accessed on 10 December 2025)).

#### 2.7.7. Genomic Plasticity Analysis

Prophage regions were identified using PhiSpy (v4.2.21). Genomic islands were predicted using IslandViewer 4 (https://www.pathogenomics.sfu.ca/islandviewer/ (accessed on 6 June 2026)). Clustered Regularly Interspaced Short Palindromic Repeats (CRISPR) loci, including direct repeats and spacer sequences, were detected using CRISPRCasFinder (v4.3.2). Insertion sequence (IS) elements were identified using ISEScan (v1.7.2.3), and transposable elements were predicted using TransposonPSI (http://transposonpsi.sourceforge.net/ (accessed on 6 June 2026)). The resulting data were integrated to evaluate the genomic plasticity of strain A3.

#### 2.7.8. Prediction of Virulence Genes

Putative virulence-associated genes were identified by comparing predicted protein sequences against the Virulence Factor Database (VFDB; http://www.mgc.ac.cn/VFs/ (accessed on 11 December 2025)) using Basic Local Alignment Search Tool for Proteins (BLASTP, v2.14.1). Hits meeting the criteria of E-value < 1 × 10^−5^, sequence identity ≥ 70%, and alignment coverage ≥ 70% were considered putative virulence genes.

#### 2.7.9. Prediction of Antimicrobial Resistance Genes

Antimicrobial resistance genes were identified using the Comprehensive Antibiotic Resistance Database (CARD, https://card.mcmaster.ca/ (accessed on 12 December 2025)). Predicted protein sequences were aligned against the CARD database using BLASTP (v2.14.1). Hits satisfying the thresholds of E-value < 1 × 10^−5^, sequence identity ≥ 70%, and query coverage ≥ 70% were retained as putative antimicrobial resistance genes.

### 2.8. Comparative Genomic Analysis of Strain A3 and Reference Strains

#### 2.8.1. ANI Calculation and Phylogenomic Analysis

To determine the genomic relatedness and evolutionary position of strain A3, whole-genome sequences of A3 and 12 publicly available *P. mirabilis* reference strains were included (*n* = 13). Pairwise average nucleotide identity (ANI) values were calculated using JSpeciesWS (https://jspecies.ribohost.com/jspeciesws/ (accessed on 8 June 2026)) with default parameters. The ANI similarity matrix was visualized as a heatmap using TBtools (v2.310). A whole-genome phylogenetic tree was inferred using the Integrated Prokaryotic Genomes Analysis platform (IPGA, v1.09) with default settings.

#### 2.8.2. Pangenome Analysis

Pangenome analysis to provide genomic context for strain A3 was performed on the same 13 *P. mirabilis* genomes using Roary (v3.13.0). Gene families were classified into core genes (present in 99–100% of strains), soft-core genes (95–99%), shell genes (15–95%), and cloud genes (0–15%). Functional annotation was performed against the COG database via IPGA. Pangenome and core-genome accumulation curves were generated to evaluate pangenome openness.

### 2.9. Animal Challenge Test of P. mirabilis Strain A3

All animal experimental procedures were approved by the Animal Ethics Committee of Anhui Science and Technology University (Approval No. AK2026001) and were conducted in strict accordance with institutional guidelines for the care and use of laboratory animals.

Twenty-four healthy specific pathogen-free (SPF) Kunming mice (male-to-female ratio = 1:1, body weight approximately 20 g) were purchased from Qinglongshan Biotechnology Co., Ltd. (Nanjing, China). The mice were housed in stainless steel cages under controlled environmental conditions, including an ambient temperature of 24 ± 2 °C, relative humidity of 40–70%, a 12 h light/dark cycle, and protection from direct strong light and high-frequency noise exposure.

The rationale for dose selection was as follows: (1) during the preliminary experimental phase, viable bacterial counts were determined, and the dilution yielding 30–300 colonies per plate was selected as the basis for colony enumeration; (2) a two-dose infection model (high-and low-dose groups) was designed to evaluate the pathogenicity of strain A3.

During the experiment, mice were provided with sterilized rodent chow (by irradiation) and sterile water ad libitum. Water bottles were checked regularly to prevent blockage. After an acclimatization period of approximately 7 days to minimize stress and ensure physiological stability, mice were fasted for 12 h before bacterial challenge, while water was provided ad libitum until sacrifice, to minimize the potential influence of recent food intake on histopathological evaluation.

A total of 24 mice were randomly divided into three groups (*n* = 8 per group): a high-dose group (Group A), a low-dose group (Group B), and a control group. Group A received an intraperitoneal injection of 0.2 mL bacterial suspension at a concentration of 2.22 × 10^9^ CFU/mL, while Group B received 0.2 mL of bacterial suspension at 2.22 × 10^8^ CFU/mL. The control group was injected intraperitoneally with an equal volume of sterile saline.

Clinical signs and mortality were monitored and recorded for 48 h post-injection. Observations were performed at least hourly during the first 12 h after infection. Mice that died or became moribund were immediately necropsied, and heart, liver, spleen, and kidney tissues were collected for bacterial re-isolation and identification.

Tissue samples were submitted to Wuhan Servicebio Technology Co., Ltd. (Wuhan, China) for histological processing, including paraffin embedding, sectioning, and hematoxylin and eosin (H&E) staining, to assess pathological changes. At the end of the experiment, surviving mice were humanely euthanized via cervical dislocation under ethical approval, followed by necropsy and collection of relevant organs. All collected samples were properly preserved for subsequent analyses.

### 2.10. Statistical Data Processing

Unless otherwise stated, experiments involving phenotypic characterization, biochemical identification, antimicrobial susceptibility testing, PCR detection, and whole-genome analysis were performed using the single bovine-derived *P. mirabilis* isolate A3; therefore, the biological replicate number for these isolate-level analyses was *n* = 1 isolate. For the mouse pathogenicity experiment, 8 mice per group were used in test group A, test group B, and the control group. The number of biological replicates for each dataset is indicated in the corresponding table notes, where applicable.

Quantitative phenotypic assays were performed in triplicate unless otherwise stated. Antimicrobial susceptibility testing was independently repeated 3 times, and inhibition zone diameters were recorded as the mean ± standard deviation (SD). The susceptibility categories of strain A3 were interpreted according to the CLSI M100 guidelines. Growth curve assays were conducted in three independent experiments, and OD600 values were expressed as means ± SD.

For the mouse pathogenicity assay, clinical signs, mortality, necropsy findings, bacterial re-isolation results, and histopathological changes were analyzed descriptively. Because this experiment was designed to evaluate the pathogenic potential of a single bovine-derived *P. mirabilis* isolate rather than to compare different treatment groups or strains, no inferential statistical tests were performed for survival or pathological outcomes.

Genome assembly, annotation, resistance gene prediction, virulence gene screening, mobile genetic element analysis, comparative genomic analysis, and pangenome analysis were performed using the bioinformatics tools and parameters described in Section 2.7 and Section 2.8. Descriptive summaries were generated for genome size, GC content, predicted coding sequences, antimicrobial resistance genes, virulence-associated genes, and comparative genomic features. No additional statistical hypothesis testing was applied to these bioinformatics results.

Data organization and basic calculations were performed using Microsoft Excel. Graphs and visualizations were generated using GraphPad Prism v9.0, TBtools v2.310, and Circos v0.69-9, as appropriate.

## 3. Results

### 3.1. Bacterial Isolation and Identification

**Figure 1:**
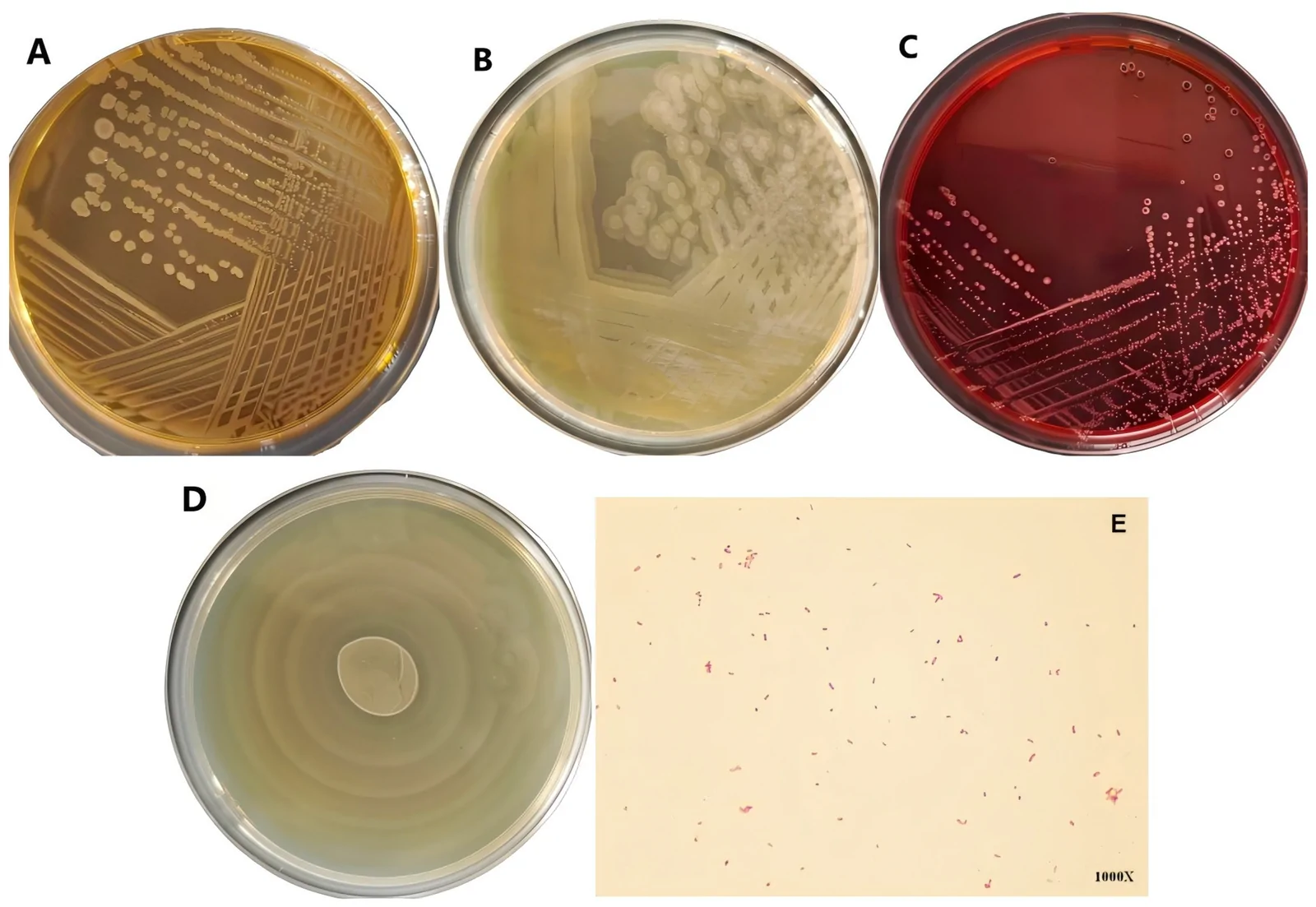
Colony morphology of *P. mirabilis* strain A3 on different media and Gram staining result. (**A**): MacConkey agar; (**B**): BHI agar; (**C**): XLD agar; (**D**): LB agar; (**E**): Gram staining microscopy (1000×).

The isolated strain exhibited characteristic colony morphologies on different culture media. On MacConkey agar, the colonies were pale yellow with brownish centers and irregular margins, and the cultures produced a characteristic fishy odor. (Figure 1A). Diffuse spreading growth was observed on BHI agar (Figure 1B). On XLD agar, the colonies appeared yellow with black centers, smooth margins, and slightly convex surfaces (Figure 1C). Characteristic swarming motility was observed on LB agar, producing concentric rings and a thin bacterial film that covered the entire agar surface (Figure 1D). The colonies were translucent, milky-white to grayish white, mucoid, and irregularly edged. Gram staining and microscopic examination (1000×) demonstrated that the isolate was a Gram-negative short bacillus (Figure 1E).

As indicated in the biochemical identification in Table 2, the results for semi-solid agar, ornithine decarboxylase broth, Simmons citrate, hydrogen sulfide (H_2_S), urease, methyl red (MR), Voges-proskauer (VP), and phenylalanine were all positive, whereas the results for lysine decarboxylase broth, amino acid decarboxylase control, peptone water (tryptophan broth), mannitol, inositol, sorbitol, melibiose, adonitol, and raffinose were all negative. Comparison of these results with the phenotypic descriptions in Bergey’s Manual of Systematic Bacteriology indicated that the isolate was consistent with *P. mirabilis*. The isolate was preliminarily identified as *P. mirabilis*.

### 3.2. 16S rRNA Gene Sequencing and Analysis

**Figure 2:**
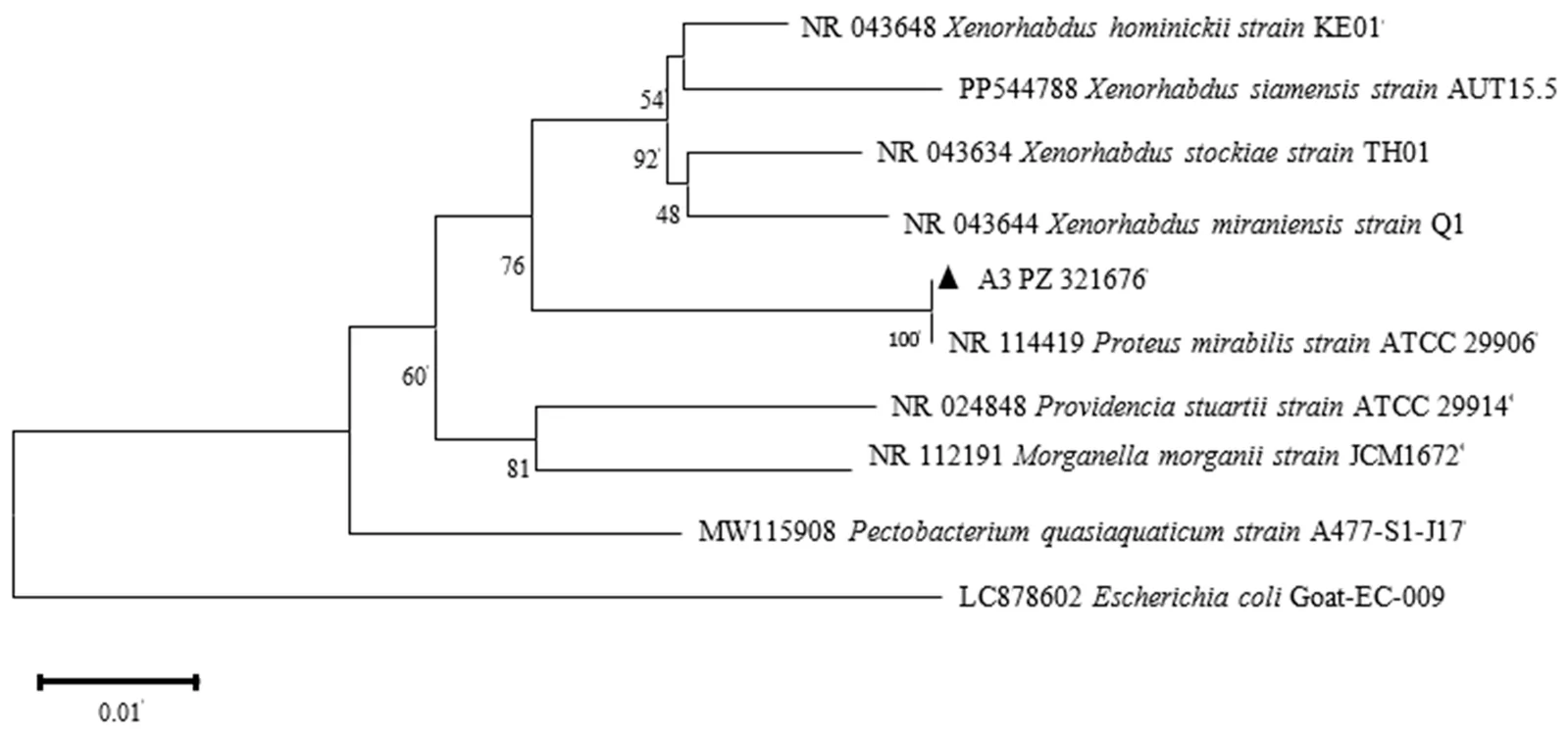
Phylogenetic tree based on the 16S rRNA gene of different bacterial strains. The *P. mirabilis* A3 isolate in this study is indicated with a solid black triangle.

The nearly full-length 16S rRNA gene sequence of strain A3 was approximately 1500 bp. BLAST analysis showed 99.73% sequence similarity to *P. mirabilis* ATCC 29906 (accession number NR 114419). A phylogenetic tree was constructed using MEGA software (version 11.0.3) based on the 16S rRNA gene sequences of strain A3 and reference strains. The phylogenetic tree (Figure 2) showed that strain A3 clustered on the same branch as *P. mirabilis* ATCC 29906. Therefore, the isolated strain A3 was identified as *P. mirabilis*.

### 3.3. Growth Curve of Strain A3

**Figure 3:**
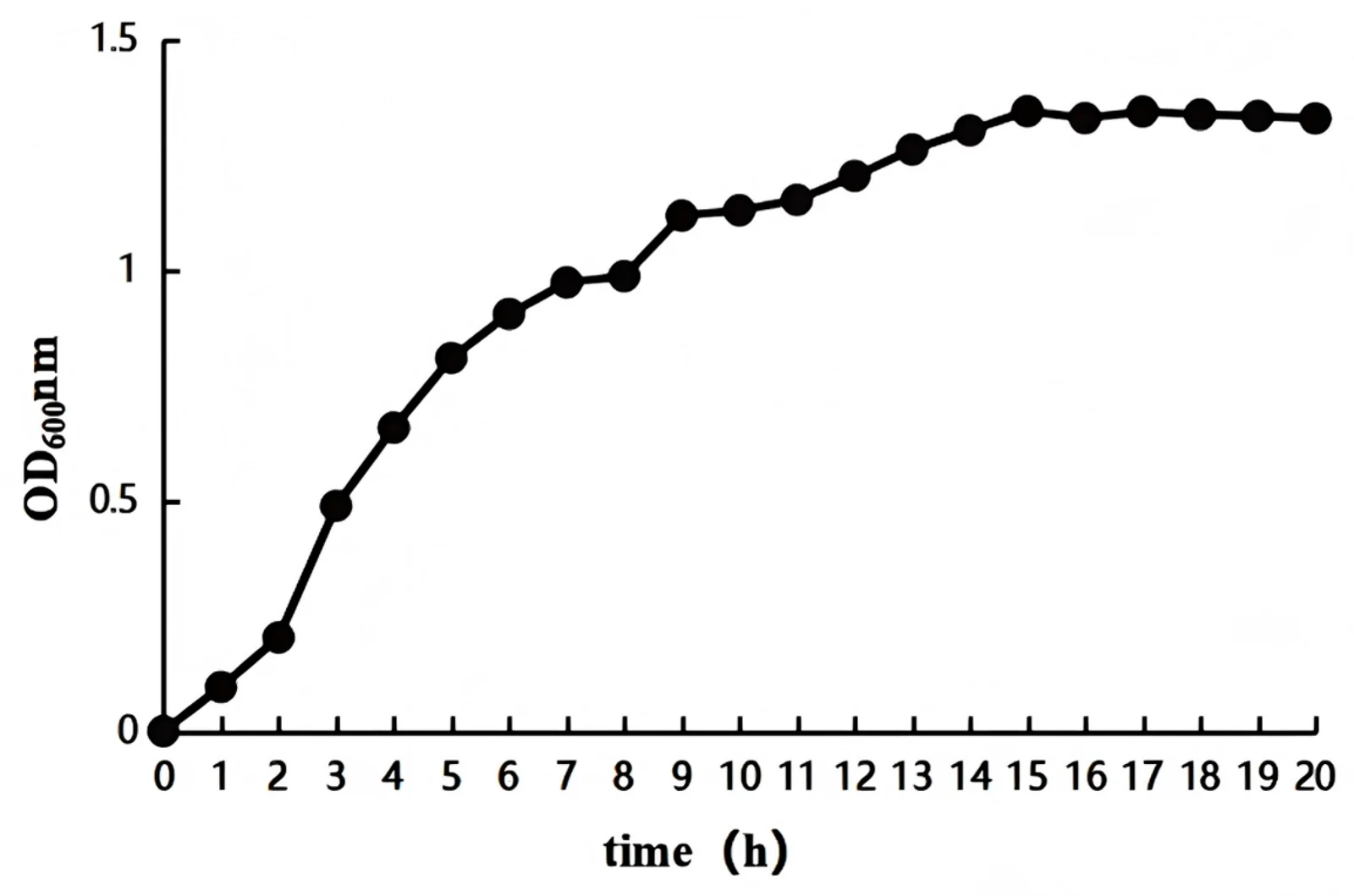
Growth curve of *P. mirabilis* strain A3.

As shown in Figure 3, strain A3 grew slowly during the first 0–2 h, then entered a rapid logarithmic growth phase. After a slow-growth period during the first 2 h, strain A3 entered the exponential phase, which lasted until approximately 15 h. Growth subsequently plateaued between 15 and 20 h.

### 3.4. Antimicrobial Susceptibility Test of Strain A3

As shown in Table 3, of the 26 antibiotics tested, strain A3 was susceptible to 13, with the largest inhibition zones observed for ceftriaxone and norfloxacin (≥30 mm). The strain is resistant to ampicillin, cefradine, imipenem, midecamycin, and tetracycline, and shows intermediate sensitivity to neomycin, minocycline, and erythromycin, indicating a multidrug-resistant phenotype. According to the 2012 international consensus definition, the isolate was classified as multidrug resistant because it was nonsusceptible to at least one agent in three or more antimicrobial categories [32].

### 3.5. Detection of Resistance and Virulence Genes of Strain A3

**Figure 4:**
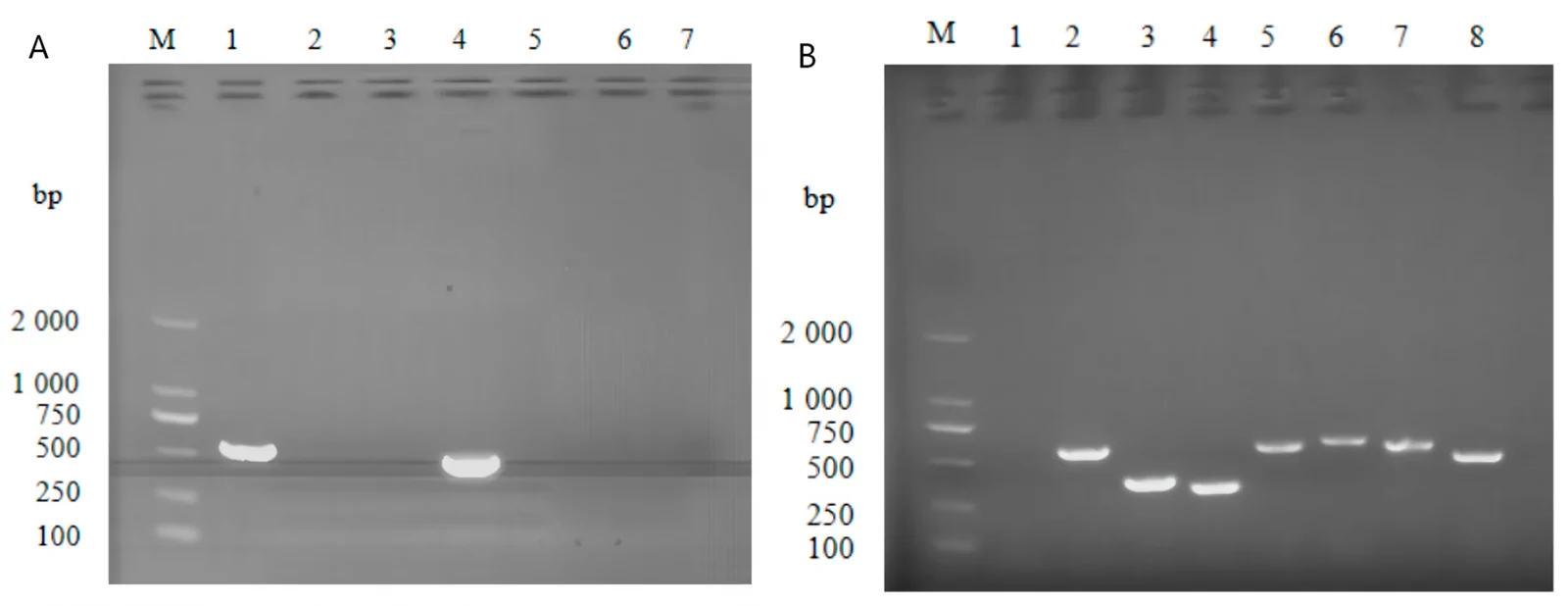
PCR amplification of antimicrobial resistance and virulence-associated genes in the *P. mirabilis* strain A3. (**A**): Antimicrobial resistance genes; M: DNA marker; 1: *aac-Ib*; 2: *qnrS*; 3: *qnrB*; 4: *qnrA*; 5: *SHV*; 6: *CTX-M*; 7: *TEM.* (**B**): Virulence-associated genes; M: DNA marker; 1: *rsbA*; 2: *pmfA*; 3: *atfA*; 4: *ureC*; 5: *mrpA*; 6: *ucaA*; 7: *zapA*; 8: *atfC*.

PCR detection of antimicrobial resistance genes in strain A3 is shown in Figure 4A. Of the seven genes examined, *aac-Ib* and *qnrA* were detected, whereas the remaining five genes were not detected. PCR detection of virulence-associated genes is shown in Figure 4B. Seven of the eight genes examined-*pmfA*, *atfA*, *ureC*, *mrpA*, *ucaA*, *zapA*, and *atfC*-were detected, whereas *rsbA* was not detected.

### 3.6. Genome Analysis Results of P. mirabilis Strain A3

#### 3.6.1. Overview of Genome Data

Strain A3 was submitted to Chuzhou General Biotechnology Co., Ltd. for whole-genome sequencing. The genome coverage statistics for strain A3 were as follows: 7,467,388 reads (1,127,575,588 bp), with an N rate of 0.0462, Q20 of 99.45%, Q30 of 97.37%, and an average depth of 278×.

#### 3.6.2. Whole-Genome Assembly

The whole-genome sequencing data for strain A3 were deposited in the NCBI database under accession number JBTMZL000000000. Detailed information is provided in Table 4. Comparison against the NT database showed that scaffold 1 shared multiple high-identity matches with *P. mirabilis* strain CP021852.1, with nucleotide identities ranging from 98.172% to 99.240% and E-values of 0.

#### 3.6.3. Genome Map Analysis

**Figure 5:**
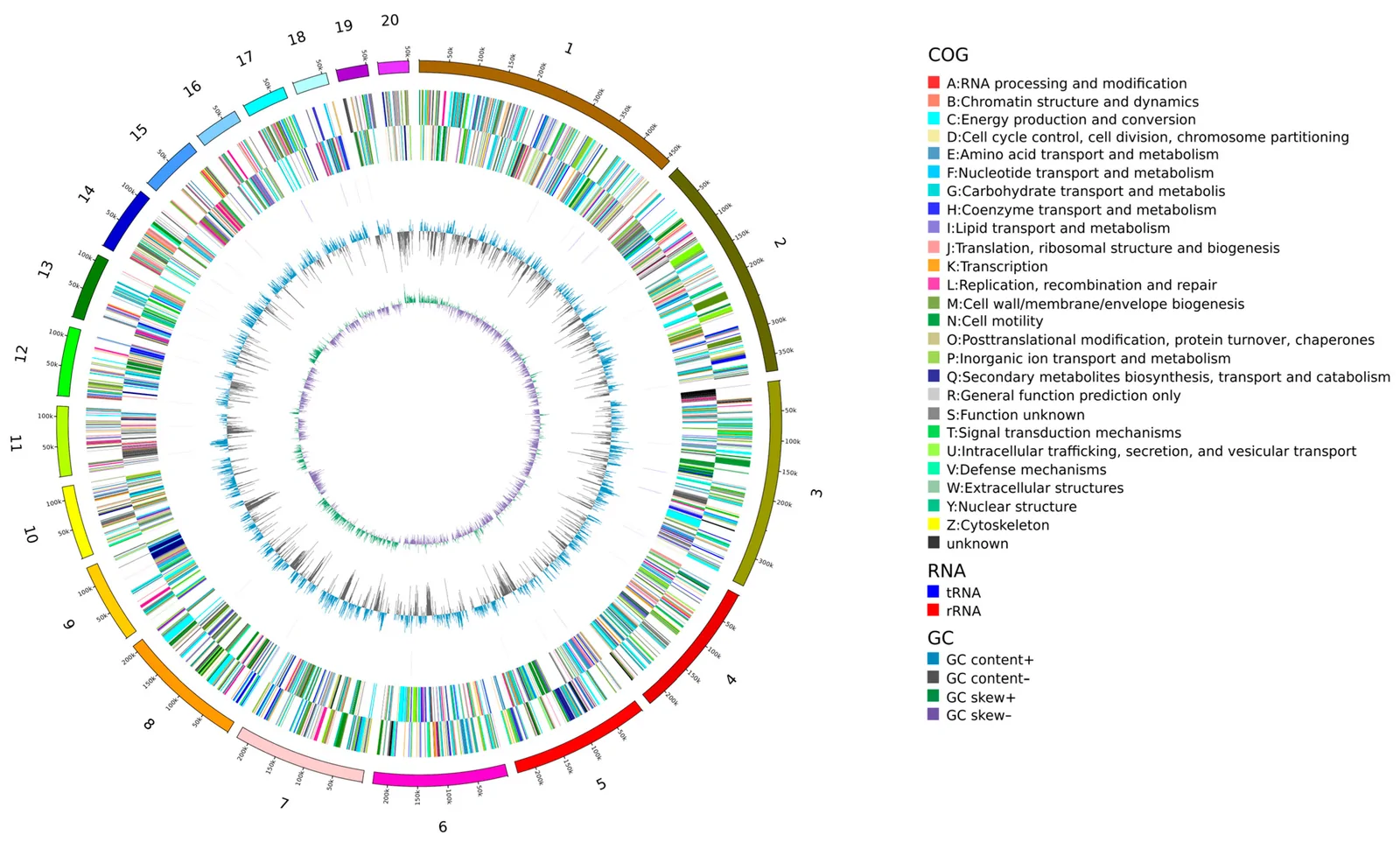
Genome map of *P. mirabilis* strain A3.

Using Circos software, a circular genome map was drawn for the top 20 scaffolds with sequence lengths greater than 5000 bp (Figure 5). From the outermost to the innermost rings, the map displays scaffold coordinates, CDSs on the forward and reverse strands colored by COG functional category, tRNA genes, rRNA genes, GC content, and GC skew. CDSs were distributed across both strands without obvious strand-specific enrichment. Genes assigned to energy production and conversion, transcription, carbohydrate transport and metabolism were broadly distributed, whereas genes assigned to RNA processing and modification and extracellular structures were less abundant. The innermost ring represents GC skew, calculated as (G − C)/(G + C).

#### 3.6.4. GO Annotation Analysis

**Figure 6:**
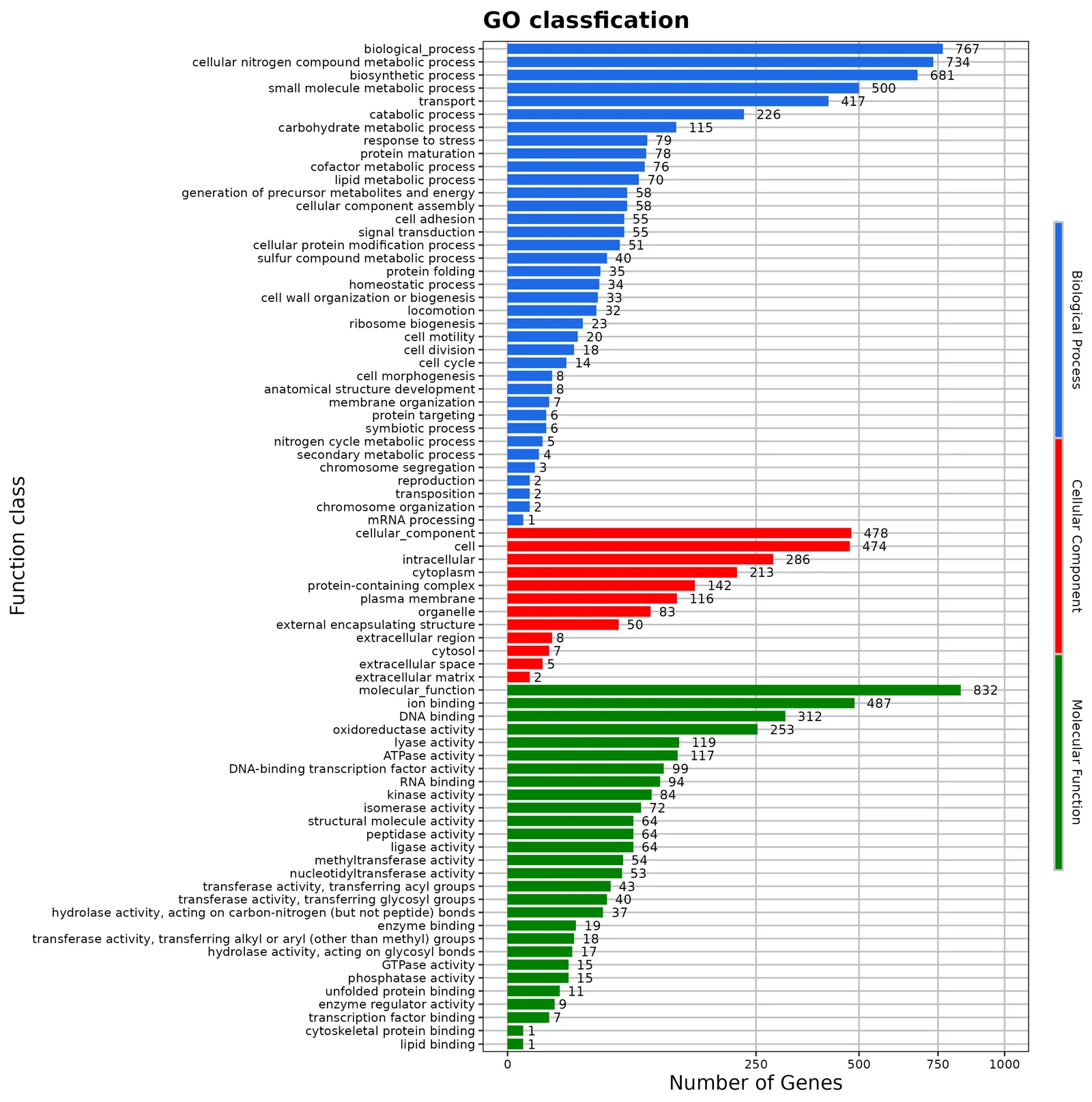
GO annotation analysis of *P. mirabilis* strain A3.

GO database annotation classified the strain’s genes into three main categories: molecular function (MF), cellular component (CC), and biological process (BP). The GO annotation results of strain A3 are shown in Figure 6.

#### 3.6.5. COG Annotation Analysis

**Figure 7:**
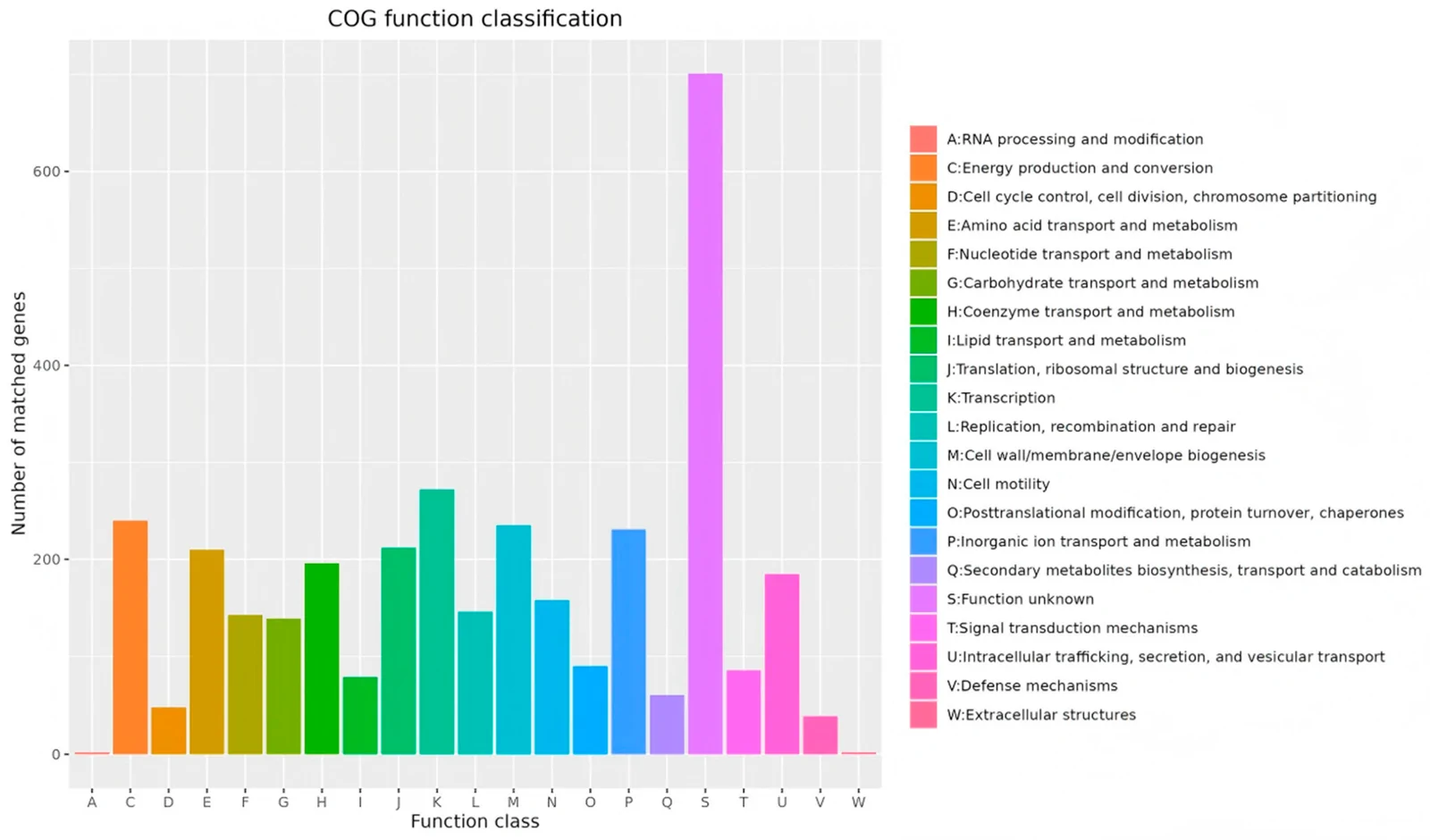
COG annotation analysis of *P. mirabilis* strain A3.

COG annotation of the functional protein-coding genes in the genome of strain A3 revealed a total of 3475 functional genes (Figure 7). The distribution was as follows: transcription (272, 7.5%), energy production and conversion (240, 6.6%), cell wall/membrane/envelope biogenesis (235, 6.5%), inorganic ion transport and metabolism (231, 6.4%), translation, ribosomal structure and biogenesis (212, 5.9%), amino acid transport and metabolism (210, 5.8%), carbohydrate transport and metabolism (139, 3.8%). Additionally, 701 genes had unknown functions.

#### 3.6.6. KEGG Annotation Analysis

**Figure 8:**
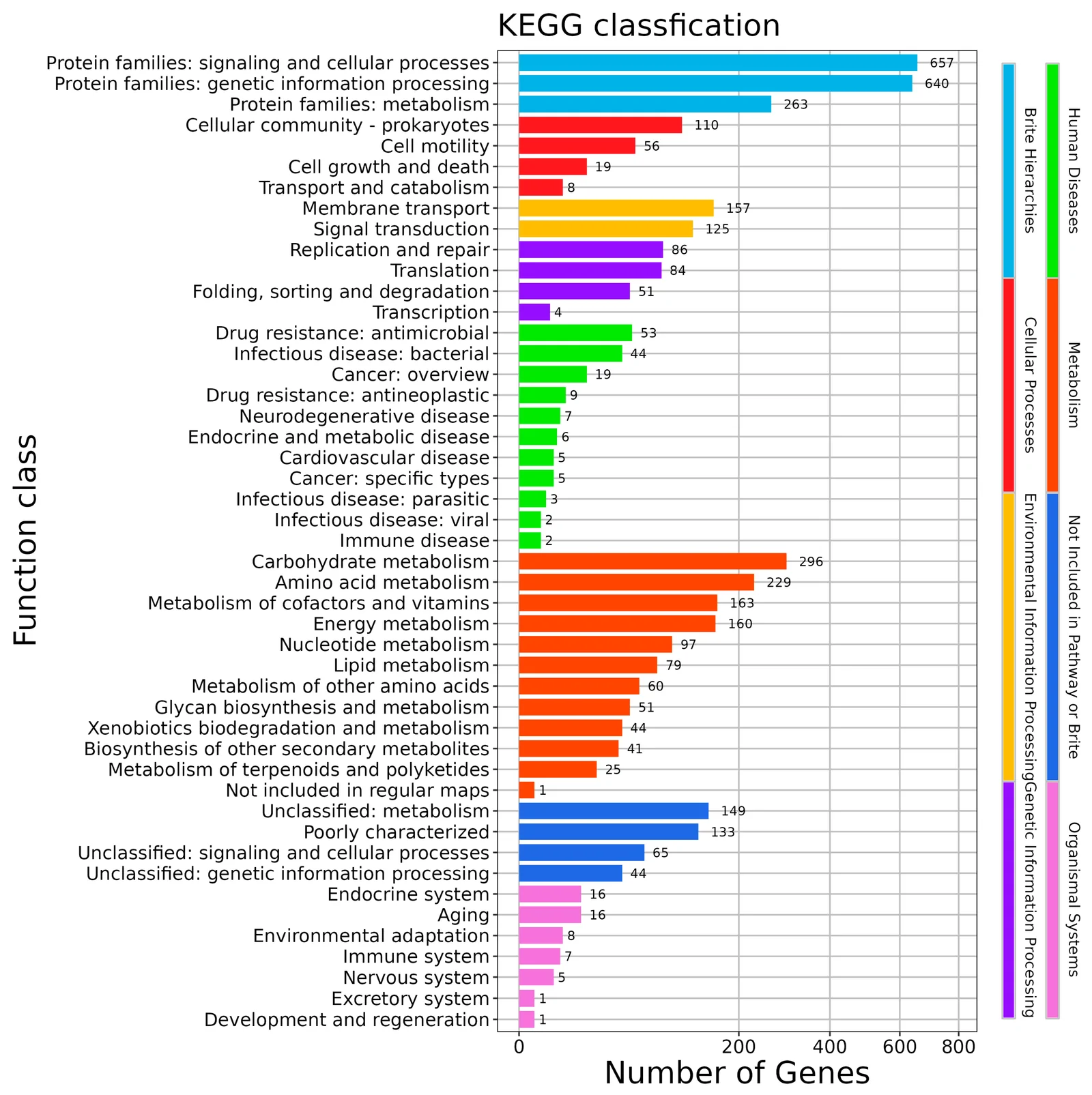
KEGG annotation analysis of *P. mirabilis* strain A3.

Among the 3637 protein-coding genes identified in strain A3, a total of 4106 KEGG annotations were assigned (Figure 8). The number of annotations exceeded the number of genes because some genes were associated with multiple KEGG orthologs and biological pathways. Genes classified under “Metabolism” were the most abundant, with 1246 genes (35.95%). Among these, carbohydrate metabolism had the highest number (296, 23.8% of metabolism), followed by amino acid metabolism (229, 18.4%), cofactor and vitamin metabolism (163, 13.1%), and nucleotide metabolism (97, 7.8%).

#### 3.6.7. Plasticity Analysis

Genomic plasticity analysis revealed multiple mobile genetic elements in the genome of *P. mirabilis* strain A3 (Table 5). A total of 12 insertion sequences (ISs) belonging to five families, namely IS200/IS605, IS21, IS3, IS481, and ISNCY, were identified, with a combined length of 16,344 bp. Among these, IS21 family elements located on scaffolds 11, 3, and 7 contained intact transposase genes and characteristic inverted repeats, suggesting that they may retain transposition potential.

Prophage analysis identified complete or partial prophage regions with a combined estimated length of approximately 567 kb, all of which contained putative attL and attR attachment sites, indicating potential integration into the host genome. Virulence factor annotation using the VFDB database revealed that several prophages harbored virulence-associated genes, with the prophage pp15 carrying more than 10 such genes. Notably, no antimicrobial resistance genes annotated in the CARD database were detected within any prophage region.

A total of 13 genomic islands were predicted, with a cumulative length of approximately 102 kb. However, no virulence- or resistance-related genes were identified within these regions. CRISPR analysis detected five CRISPR loci distributed across scaffolds 2, 3, 5, 8, and 18, among which the locus on scaffold 5 contained the highest number of spacers (*n* = 7).

Homology-based transposon analysis identified four ISC1316-related fragments and one putative LINE-like element. All of these elements were fragmented and exhibited low sequence similarity to known transposable elements.

#### 3.6.8. Resistance Gene Annotation

Genome annotation identified multiple resistance-associated genes in strain A3, including genes encoding multidrug efflux systems (Table 6). Several annotated efflux-associated genes, including acrB, components of the MexAB system, and tolC, have been associated with reduced susceptibility to multiple antimicrobial classes. Among the 85 resistance-associated genes identified, efflux pump-related genes accounted for a high proportion, with RND-family efflux pump genes being the most frequently annotated, suggesting that efflux may contribute to the resistance potential of strain A3.

#### 3.6.9. Virulence Gene Annotation

A total of 147 virulence-associated genes were detected in the genome of strain A3 (Table 7). Among them, adhesion genes (33) and motility genes (32) were the most abundant, followed by immune modulation genes (27) and nutritional/metabolic factor genes (29). Effector delivery system genes (11) were also present. Strain A3 possesses MR/P, PMF, and UCA fimbrial systems, which may facilitate adhesion to different host tissues, including uroepithelial surfaces. These systems are well-characterized virulence-associated features in uropathogenic *P. mirabilis*. Motility-related genes included complete sets for flagellar assembly, motor proteins, and chemotaxis systems. Nutritional/metabolic factors were primarily involved in iron acquisition from the host. Immune modulation genes were mainly associated with lipopolysaccharide (LPS) and lipooligosaccharide (LOS) biosynthesis. Genes encoding components of the type VI secretion system, including *hcp*, *vgrG3*, and *clpV*, were identified. The hemolysin genes *hpmA* and *hpmB* and the metalloprotease gene *zapA* were also detected.

### 3.7. Comparative Genomic Analysis of Strain A3 and Reference Strains

#### 3.7.1. ANI-Based Genomic Relatedness and Phylogenomic Relationships

**Figure 9:**
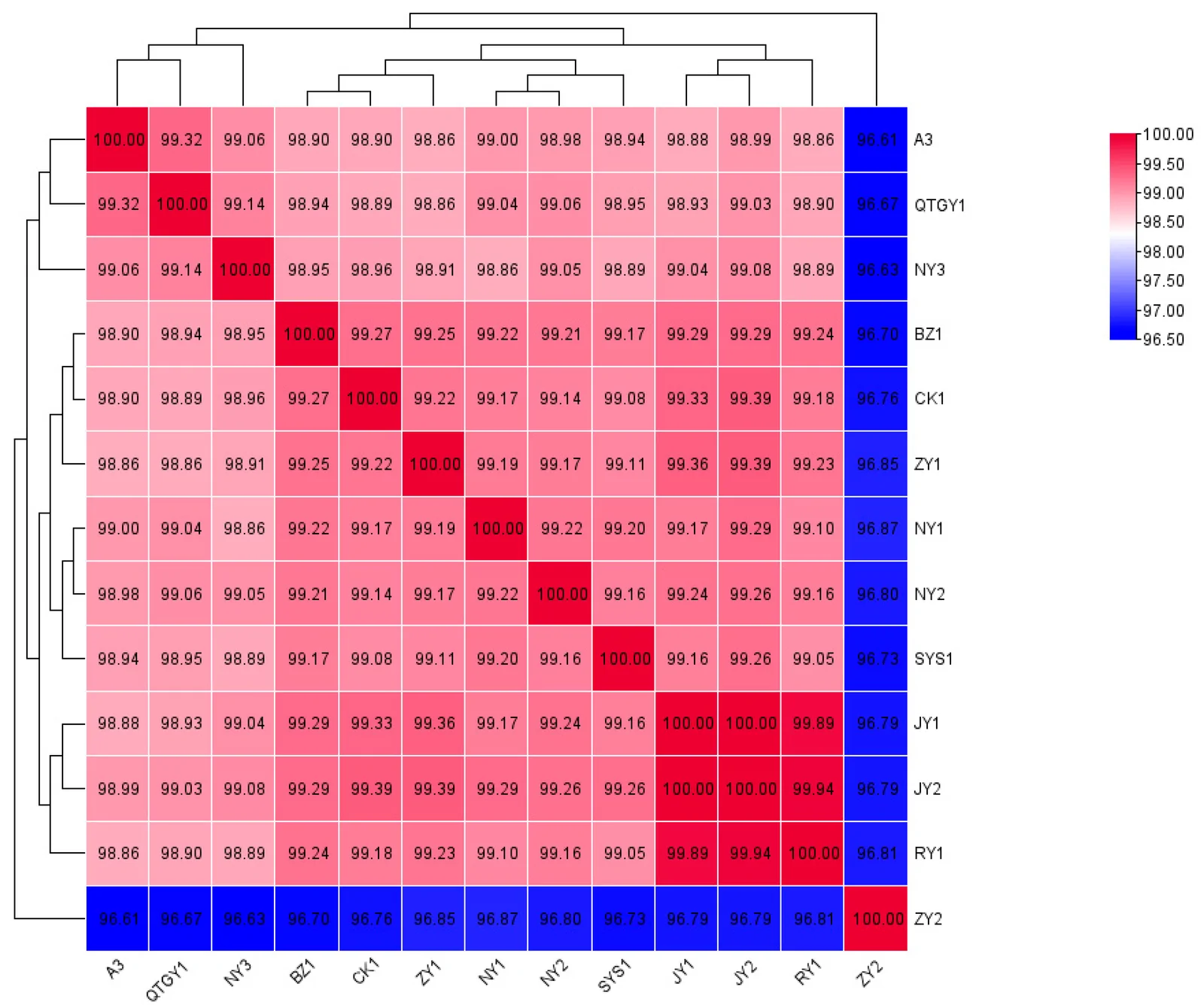
Heatmap of average nucleotide identity values among 13 *P. mirabilis* strains. Note: The heatmap shows the pairwise ANI values among strain A3 and 12 reference *P. mirabilis* strains. Warmer colors indicate higher genomic similarity, and the values within each cell represent the corresponding ANI percentages.

To confirm the taxonomic status of strain A3 at the whole-genome level and assess its evolutionary relatedness to strains from different hosts, ANI analysis was performed between A3 and 12 reference *P. mirabilis* genomes (Figure 9). All pairwise ANI values between A3 and the reference *P. mirabilis* strains were above 95%, meeting the accepted species delineation threshold for prokaryotes. Notably, A3 showed the highest ANI values with bovine-derived strains (NY1, NY2, and NY3), ranging from 99.06% to 99.32%, indicating very close intraspecies relatedness. In contrast, strain ZY2 (porcine origin) showed relatively lower ANI values (~96.6–96.9%) than the other strains, but remained within the expected intraspecies range.

**Figure 10:**
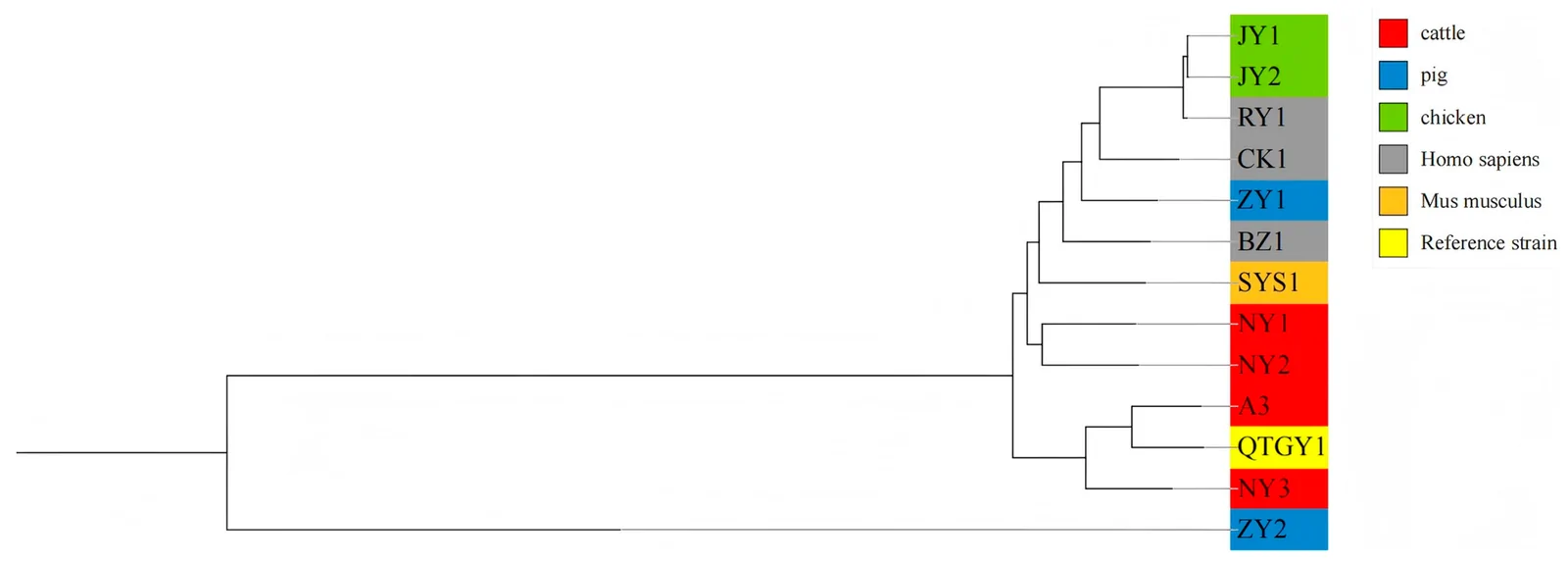
Whole-genome phylogenetic tree of 13 *P. mirabilis* strains. Note: The phylogenetic tree was constructed based on whole-genome sequences. Branch values indicate confidence levels. Colors on the right represent the host origins of the strains: red, cattle; green, chicken; blue, pig; yellow, reference strain; orange, laboratory mouse; gray, human.

The bovine-derived strains NY1, NY2, NY3, and A3 tended to cluster; however, they did not form a strictly host-specific monophyletic group (Figure 10). This finding suggests that intraspecies genomic diversification in *P. mirabilis* is not strictly associated with host origin. The porcine strain ZY2 formed an early-diverging branch, consistent with its relatively lower ANI values.

#### 3.7.2. Pangenome Analysis of 13 *P. mirabilis* Genomes

Pangenome analysis was performed using 13 *P. mirabilis* genomes, including strain A3, to evaluate genomic diversity and evolutionary characteristics. A total of 5680 orthologous gene clusters were identified, comprising 2959 core genes, 110 soft-core genes, 1200 shell genes, and 1400 cloud genes. These results indicate that more than half of the gene repertoire is conserved across strains, whereas shell and cloud genes contribute substantially to intraspecies genomic diversity.

**Figure 11:**
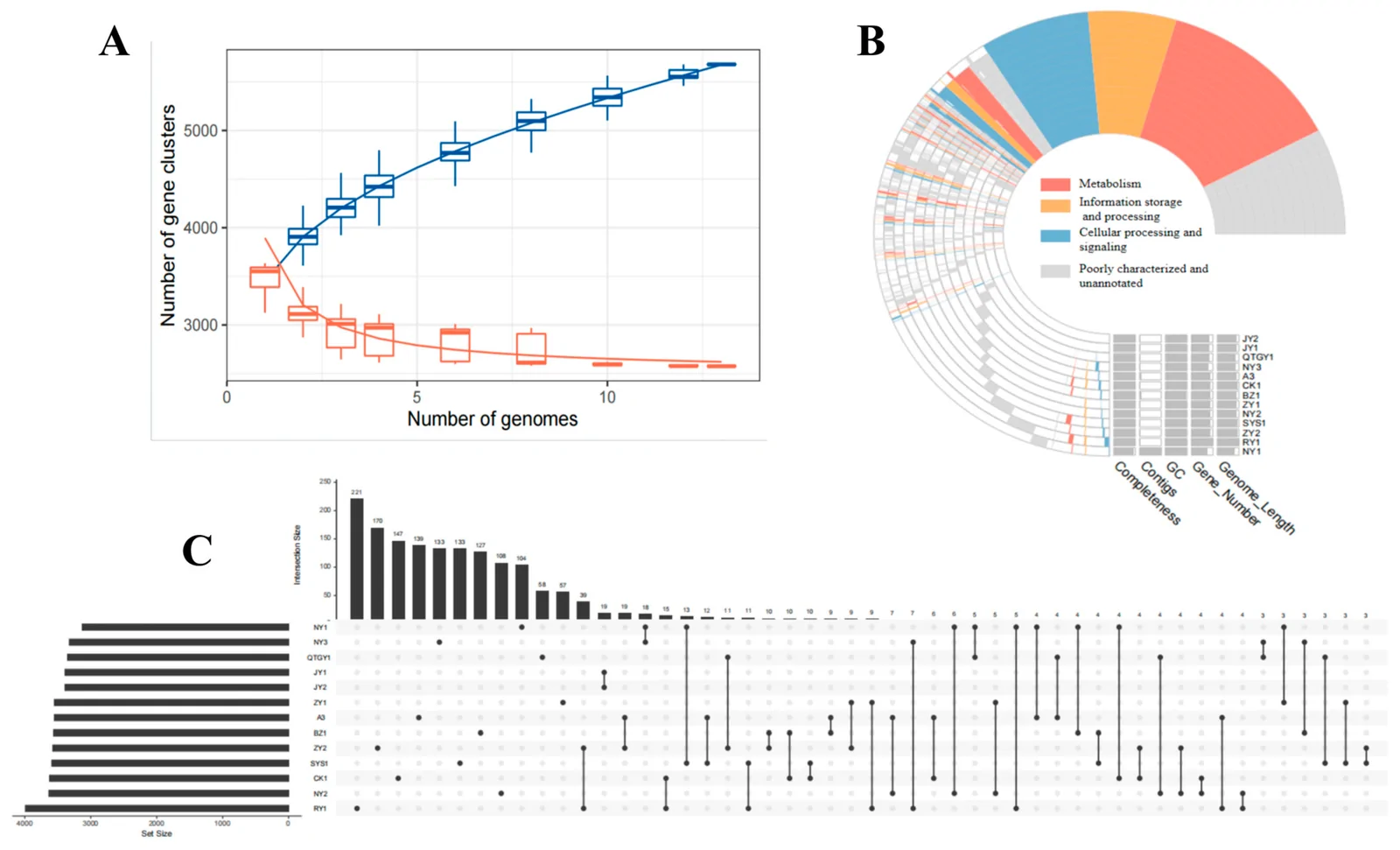
Pangenome analysis of 13 *P. mirabilis* strains. Note: (**A**) Core-genome and pangenome accumulation curves. The blue curve represents the pangenome size, and the red curve represents the core-genome size. (**B**) COG functional annotation profile showing the functional classification of pangenome components. Different colors represent COG functional categories; the outer rings indicate genome features, such as gene number and GC content, for each strain. (**C**) Strain-specific gene cluster analysis. The lower panel shows the distribution of shared gene clusters among strains, and the upper bar chart indicates the number of strain-specific gene clusters for each strain.

As shown in Figure 11A, the size of the pangenome continued to increase with the sequential addition of genomes and did not reach a plateau, while the number of core genes gradually decreased to approximately 2959 genes across the 13 genomes analyzed. These findings suggest that *P. mirabilis* possesses an open pangenome, reflecting considerable genomic plasticity and a continuous capacity for gene acquisition during evolution.

Functional annotation based on the COG database revealed that pangenome genes were distributed across a broad range of functional categories (Figure 11B). Core genes were mainly associated with essential cellular functions, including transcription, translation, energy production and conversion, amino acid transport and metabolism, and cell wall/membrane biogenesis. In contrast, accessory genes were enriched in functions related to environmental adaptation, defense mechanisms, signal transduction, and mobile genetic elements, highlighting their potential roles in niche adaptation and genomic diversification.

Analysis of gene cluster distribution among the 13 strains demonstrated substantial genomic variation within the species (Figure 11C). No strain-specific gene family was identified in strain A3, indicating a high degree of gene content similarity with the other *P. mirabilis* genomes included in this study. In contrast, strain ZY2 exhibited distinct presence-absence patterns in several gene families, which may reflect host-associated adaptation and evolutionary divergence. Collectively, these results further illustrate the extensive genomic diversity and adaptive potential of *P. mirabilis*.

### 3.8. Animal Pathogenicity Test of P. mirabilis Strain A3

Mice in test group A showed clinical signs, including loss of appetite, depression, and piloerection, 2 h after artificial infection, and all died within 12 h. Mice in test group B showed similar clinical signs after 4 h, with one death within 12 h; the remaining 7 mice survived. No abnormalities were observed in the control group.

#### Histopathological Observation of Dead Mice After Challenge

**Figure 12:**
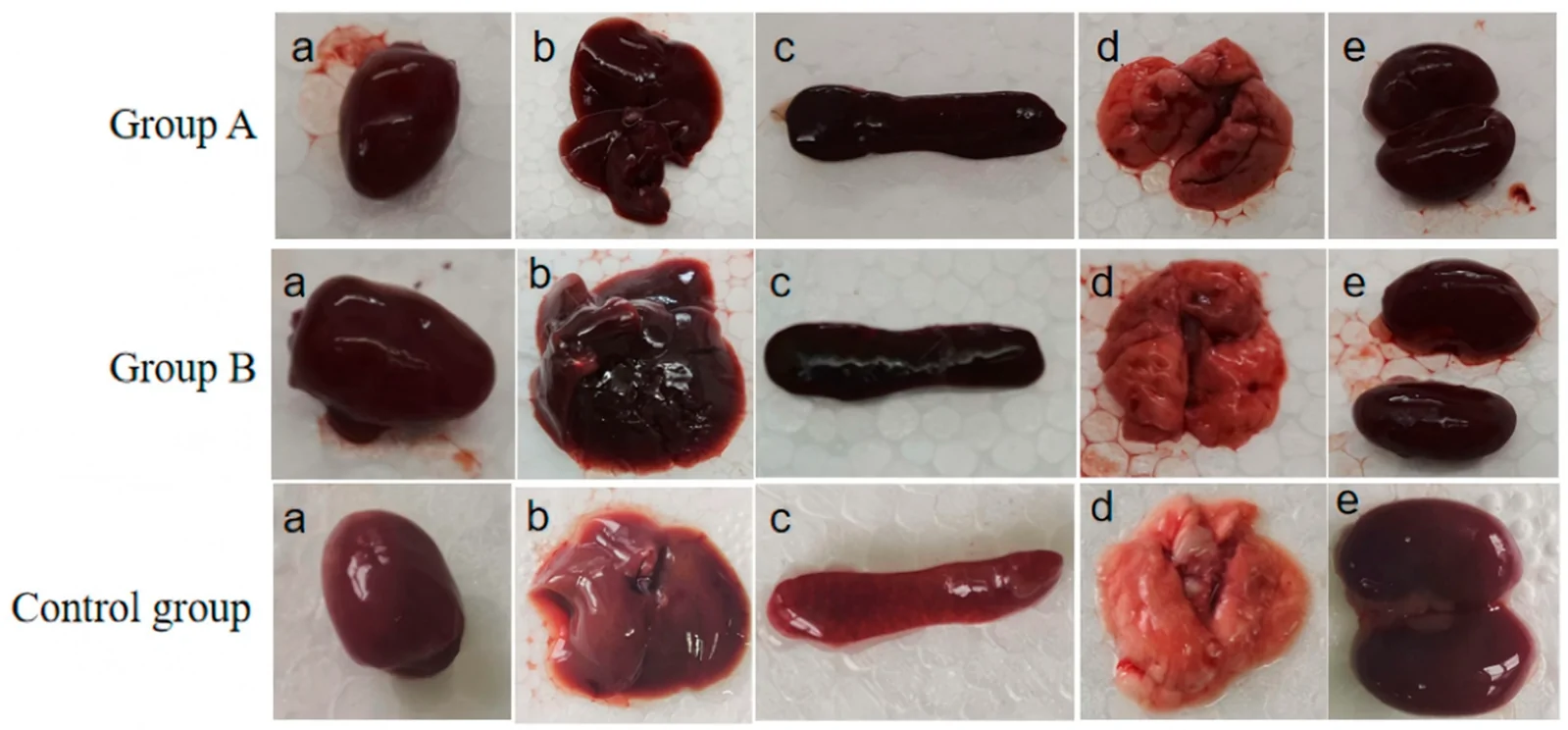
Gross pathological changes in organs from mice that died following challenge with *P. mirabilis* strain A3. (**a**): heart; (**b**): liver; (**c**): spleen; (**d**): lung; (**e**): kidney.

Necropsy of the deceased mice in groups A and B revealed varying degrees of damage in the heart, liver, spleen, lungs, and kidneys, with obvious damage observed in the liver and spleen, and varying severity of damage in the heart, lungs, and kidneys. *P. mirabilis* was re-isolated in various tissues. The histological changes in mice following inoculation with *P. mirabilis* A3 are shown in Figure 12.

**Figure 13:**
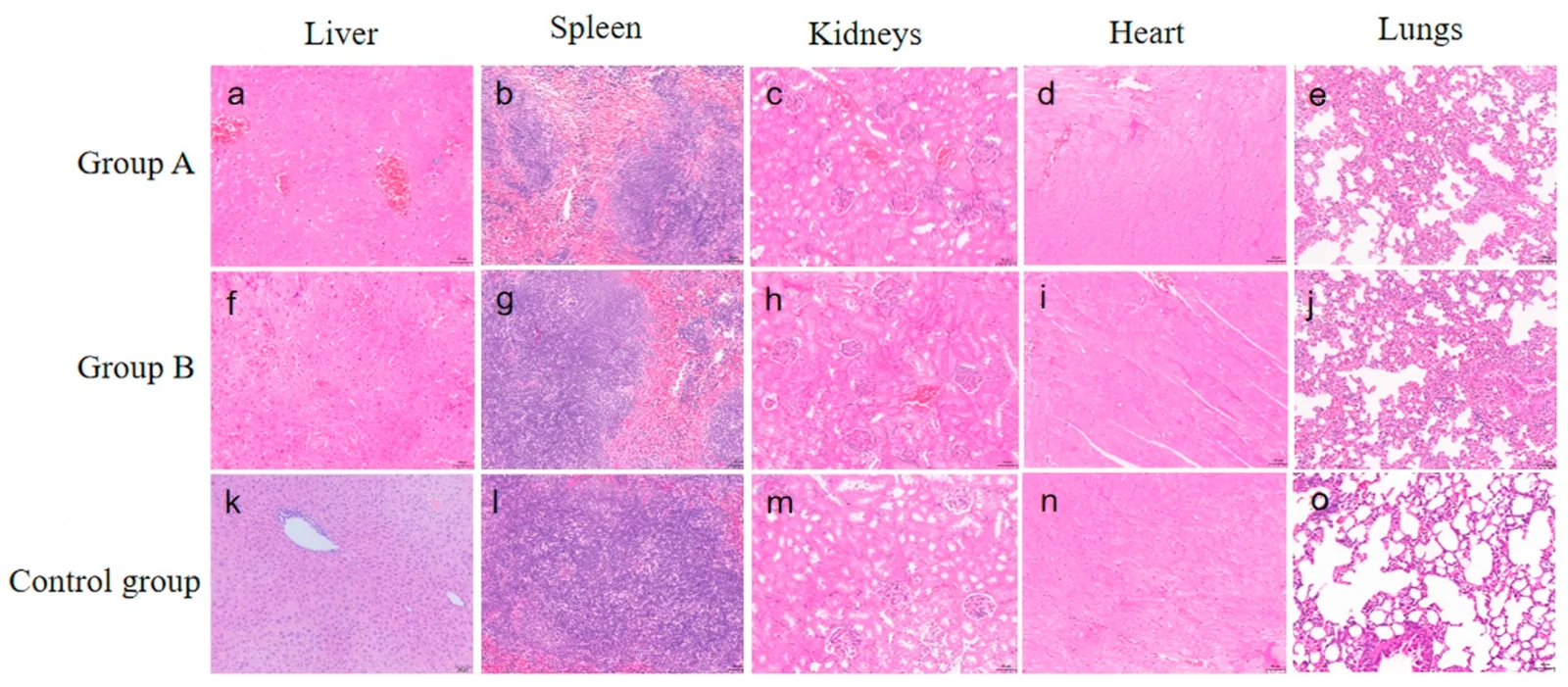
Histopathological sections of mice that died from *P. mirabilis* A3 infection. (**a**): Group A liver; (**b**): Group A spleen; (**c**): Group A kidney; (**d**): Group A heart; (**e**): Group A lung; (**f**): Group B liver; (**g**): Group B spleen; (**h**): Group B kidney; (**i**): Group B heart; (**j**): Group B lung; (**k**): Control liver; (**l**): Control spleen; (**m**): Control kidney; (**n**): Control heart; (**o**): Control lung.

Heart: In group A (Figure 13d) and group B (Figure 13i), the myocardial tissue structure was basically intact, with only local, scattered pathological changes. A few myocardial cells showed hydropic degeneration, characterized by increased cell volume and loose, unevenly stained cytoplasm. A few lipid droplets were present in the cytoplasm of some myocardial cells, with local inflammatory infiltration. The myocardial interstitium was widened. No extensive necrosis was observed.

Liver: In group A (Figure 13a), marked congestion was observed in the central vein and portal vein areas of the hepatic lobules. Some hepatic sinusoids were mildly dilated with congestion. A few lipid droplets were present in the cytoplasm, along with obvious inflammatory infiltration. In group B (Figure 13f), the arrangement of hepatic cell cords was irregular, suggesting hepatocyte damage. Some hepatic sinusoids were slightly dilated with mild congestion. Numerous lipid droplets of varying sizes were present in the cytoplasm, accompanied by mild inflammatory infiltration.

Spleen: In group A (Figure 13b), the main manifestation was obvious hemorrhagic lesions. Obvious hemorrhage and congestion were observed in both white and red pulp. Erythrocyte infiltration was visible in the white pulp, along with scattered punctate necrotic foci. Megakaryocytes were significantly reduced in the spleen. In group B (Figure 13g), splenic congestion and hemorrhage were observed, manifested by dilation of the red pulp sinuses filled with numerous erythrocytes. A few megakaryocytes were pyknotic and necrotic.

Lungs: In group A (Figure 13e), the lung tissue structure was abnormal, with alveolar collapse and thickening of the alveolar walls. Surrounding alveoli showed fusion and dilation, with no obvious fat vacuoles. Type II alveolar cells were slightly increased, and a small amount of inflammatory infiltration was observed around the bronchi and in the alveolar interstitium. Marked dilation and congestion of alveolar wall capillaries were observed. In group B (Figure 13j), the lung tissue structure was slightly abnormal, with mild alveolar collapse. A small amount of inflammatory infiltration was observed around the bronchi and in the alveolar interstitium. Alveolar wall capillaries were dilated and congested.

Kidneys: In group A (Figure 13c), renal glomerular interstitial congestion, proliferation of parietal epithelial cells forming crescentic bodies, increased volume of renal tubular epithelial cells with eosinophilic cytoplasm, irregular tubular lumina, and necrosis of some cells were observed. Proximal convoluted tubules showed edema. Inflammatory cell infiltration was present. In group B (Figure 13h), renal glomerular interstitial congestion, a slight increase in volume, and increased cell number were observed. Some glomerular capsules showed thickened walls. The glomerular capsule space was narrowed. Proximal convoluted tubules showed edema. Mild inflammatory cell infiltration was present.

## 4. Discussion

*P. mirabilis* is an important opportunistic pathogen associated with urinary tract infections, bacteremia, and infections in both humans and animals [33,34]. In recent years, increasing attention has been paid to its role in animal-derived diarrheal cases, particularly in mixed or secondary infections involving calves [35]. However, information regarding bovine-derived *P. mirabilis* in China remains limited. In the present study, a *P. mirabilis* strain designated A3 was isolated from diarrheic beef cattle in Anhui Province and characterized using phenotypic assays, antimicrobial susceptibility testing, PCR detection of resistance and virulence genes, whole-genome analysis, comparative genomics, and a mouse infection model.

Strain A3 showed typical phenotypic and biochemical characteristics of *P. mirabilis*. Its colony morphology, Gram-negative, short-rod appearance, biochemical profile, and 16S rRNA gene sequence similarity of 99.73% to *P. mirabilis* ATCC 29906 supported its identification as *P. mirabilis*. These findings confirmed that strain A3 was a bovine-derived *P. mirabilis* isolate and provided the basis for further investigation of its resistance, virulence, genomic features, and pathogenic potential.

Antimicrobial susceptibility testing showed that strain A3 was resistant to ampicillin, cefradine, imipenem, midecamycin, and tetracycline, and showed intermediate susceptibility to neomycin, minocycline, and erythromycin, indicating a multidrug-resistant phenotype [32]. This resistance profile is generally consistent with reports of *P. mirabilis* isolates from fox, raccoon, and mink farms in Shandong Province, China [36], but differs from isolates obtained from broiler farms in the same region, which showed high resistance to ciprofloxacin and chloramphenicol [37]. Such differences may be attributable to variations in antimicrobial usage, host species, farm management practices, and local selective pressures. [36,37]. Multidrug resistance in *P. mirabilis* is generally believed to arise from the combined effects of acquired resistance genes, chromosomal determinants, altered membrane permeability, target-site variation, and multidrug efflux systems, rather than from a single mechanism alone [21,23,34]. This pattern also supports the view that *P. mirabilis* can serve as a flexible reservoir of antimicrobial resistance at the human–animal-environment interface [13,16,21,34,38]. Notably, strain A3 was resistant to ceftazidime but remained susceptible to ceftriaxone, cefoperazone, and piperacillin. This phenotype may be attributable to differential β-lactamase activity against individual cephalosporins, together with possible effects of changes in outer membrane permeability or efflux activity [39,40]. In Enterobacterales, β-lactam resistance often reflects the interaction of enzymatic and permeability-related mechanisms rather than β-lactamase production alone [39,41]. Therefore, the absence of the commonly screened ESBL genes does not rule out the involvement of other chromosomal or mobile genetic elements conferring the observed β-lactam resistance phenotype.

PCR analysis of strain A3 detected the aminoglycoside resistance gene *aac(6′)-Ib* and the plasmid-mediated quinolone resistance gene qnrA, whereas *blaTEM*, *blaCTX-M*, *blaSHV*, *qnrB*, and *qnrS* were not detected. The antimicrobial susceptibility phenotype was not fully consistent with the detected resistance genes. Strain A3 showed resistance to several β-lactam antibiotics in the absence of the commonly screened ESBL genes and remained susceptible to fluoroquinolones in the presence of *qnrA*. Such inconsistencies have been reported in *P. mirabilis* and other Enterobacterales and may reflect the combined effects of acquired resistance genes, intrinsic chromosomal determinants, regulatory pathways, efflux systems, membrane permeability, and strain-specific genomic backgrounds rather than the presence of single resistance genes alone [34,39,40,42,43]. Similar genotype-phenotype discrepancies have also been reported in studies evaluating genome-based prediction of antimicrobial resistance, where the detection of resistance-associated genes does not always correspond to phenotypic resistance, especially when gene expression, promoter context, copy number, and interactions with other mechanisms have not been examined experimentally [43].

Whole-genome analysis provided additional insights into these discrepancies. Several chromosomal resistance-associated determinants were annotated, including multidrug efflux systems such as AcrAB-TolC and other RND-family efflux components. Efflux pumps are known to contribute to low-level or intermediate resistance in Gram-negative bacteria by reducing intracellular antibiotic accumulation and by acting together with target modification, porin loss, or acquired resistance enzymes [40]. In *P. mirabilis*, as in other Enterobacterales, these combined mechanisms may broaden the spectrum of resistance and facilitate the development of multidrug resistance [21,34,40]. Efflux systems may also support bacterial adaptation and persistence under host-associated conditions, linking resistance-related traits with fitness and colonization ability [34,40]. Target-associated loci, including those encoding *PBP3*, *GyrB*, and *ParE*, were identified. The presence of these loci alone does not indicate the presence of resistance-conferring mutations, but alterations in penicillin-binding proteins, DNA gyrase, or topoisomerase IV may reduce susceptibility to β-lactams and quinolones, especially when combined with efflux activity or reduced membrane permeability [39,40,44]. Although porin expression and structural changes were not examined in this study, porin loss or remodeling has been shown to play an important role in enterobacterial resistance by restricting antibiotic influx and strengthening the effects of other resistance mechanisms [41]. The fluoroquinolone-susceptible phenotype of A3, despite the presence of qnrA, is therefore not unexpected. Plasmid-mediated *qnr* genes usually provide only low-level quinolone protection, whereas clinically relevant fluoroquinolone resistance often depends on additional chromosomal mutations in quinolone resistance-determining regions, particularly in *gyrA*, *gyrB*, *parC*, or *parE*, or on the combined action of several resistance mechanisms [44]. Collectively, these findings suggest that the multidrug-resistant phenotype of strain A3 is likely to reflect interactions among acquired resistance genes, intrinsic chromosomal determinants, multidrug efflux systems, altered membrane permeability, and other as-yet-uncharacterized factors. This finding illustrates the complexity of resistance expression in *P. mirabilis* and highlights the value of combining phenotypic susceptibility testing with whole-genome analysis [34,43].

Virulence gene analysis showed that strain A3 carried *pmfA*, *atfA*, *ureC*, *mrpA*, *ucaA*, *zapA*, and *atfC*, which is generally consistent with virulence gene profiles reported in poultry isolates from Hunan, China [45], and beef isolates from Paraná, Brazil [24]. The absence of *rsbA* differs from reports in poultry production chains in Zhejiang Province and in dairy cattle isolates from Xinjiang, China [22,46], suggesting that virulence gene distribution may vary by host source, geographic region, and epidemiological background. Whole-genome analysis further identified genes associated with adhesion, motility, chemotaxis, iron acquisition, secretion systems, hemolysin production, and protease activity. In particular, the presence of *MR/P*, *PMF*, and *UCA* fimbrial systems suggests that strain A3 may have the capacity for host cell adhesion and colonization [18,19,47]. Although these virulence determinants have been extensively studied in urinary tract infections, their biological functions, including epithelial adhesion, colonization, motility, and competition with other microorganisms, are not restricted to the urinary tract and may also facilitate persistence within the intestinal environment [1,17,47]. In addition, the complete flagellar and chemotaxis systems may facilitate bacterial movement toward favorable host microenvironments and contribute to the early establishment of infection [1,48].

*P. mirabilis* is classically recognized as a uropathogen; however, increasing evidence indicates that it may also participate in gastrointestinal disease under specific host or ecological conditions [35,49,50]. Strain A3 was isolated from an adult beef cow with mild diarrhea and carried several virulence-associated determinants commonly linked to uropathogenicity, including fimbriae, flagella, chemotaxis proteins, T6SS, hemolysin *HpmAB*, and metalloprotease *ZapA* [47,48,51,52,53,54]. These factors are primarily involved in adhesion, colonization, bacterial competition, and host tissue interaction rather than the production of classical enterotoxins. Therefore, the mild diarrheal signs observed in the naturally infected animal may have resulted from localized intestinal colonization, mucosal interaction, and inflammatory responses rather than toxin-mediated enteric disease. Because comprehensive screening for other enteric pathogens was not performed in the affected animal, the present study cannot conclusively establish that strain A3 was the sole causative agent of the diarrhea. It is also possible that *P. mirabilis* acted as an opportunistic pathogen or contributed to disease progression in combination with other unidentified factors. The isolation of strain A3 from a diarrheic animal, together with its multidrug-resistant phenotype, diverse virulence-associated determinants, and pathogenicity in the mouse infection model, supports the possibility that bovine-derived P. mirabilis may contribute to enteric disease under certain conditions. This interpretation is in agreement with previous reports describing *P. mirabilis* as a secondary or opportunistic contributor to diarrhea and enteric disorders in animals [35,49,50].

Functional genome annotation further provided insights into the biological potential of strain A3. COG classification showed that genes related to transcription, energy production and conversion, cell wall/membrane/envelope biogenesis, and inorganic ion transport and metabolism were abundant, suggesting active basal metabolism, membrane-associated transport capacity, and regulatory flexibility. KEGG analysis also indicated that strain A3 possesses pathways involved in carbohydrate metabolism, amino acid metabolism, and transport systems, which may facilitate bacterial survival and proliferation under favorable host or environmental conditions [31]. However, the relationship between these metabolic features and intestinal colonization requires further experimental validation.

Comparative genomic analysis demonstrated that strain A3 shared high genomic similarity with bovine-derived strains NY1, NY2, and NY3, with ANI values ranging from 99.06% to 99.32%. This close genomic relationship suggests a conserved genomic backbone among cattle-associated *P. mirabilis* isolates and may reflect adaptation to similar host environments. At the same time, pangenome analysis of 13 *P. mirabilis* genomes identified 2959 core genes, while shell and cloud genes accounted for a considerable proportion of the pangenome. The continued expansion of the pangenome with the addition of new genomes indicates an open pangenome structure for *P. mirabilis*, a conclusion further supported by the inclusion of strain A3, suggesting that gene gain and loss remain important drivers of intraspecies diversity and ecological adaptation [55,56]. No strain-specific gene family was identified in A3, suggesting that its pathogenic potential may be mainly associated with conserved virulence determinants rather than unique lineage-specific genes.

Mobile genetic elements further supported the genomic plasticity of strain A3. Twelve insertion sequences belonging to five families were identified, among which IS21 family elements contained intact transposase genes and typical inverted repeats, suggesting potential mobility. These elements may contribute to genome rearrangements or influence resistance-related phenotypes through regulatory disruption or gene mobilization [55,57]. Mobile genetic elements are thought to play an important part in the emergence and dissemination of antimicrobial resistance in *P. mirabilis*, particularly through insertion sequences, transposons, integrons, plasmids, and other horizontally transferable structures involved in gene capture, recombination, and spread [21,23,34,38]. Although the transferability and exact genomic context of all resistance-associated determinants in A3 were not resolved here, the presence of multiple insertion sequences suggests that this genome retains the potential for ongoing rearrangement and genetic exchange. In addition, 22 complete or partial prophage regions were predicted in the A3 genome, with a combined length of approximately 567 kb. Several prophages harbored virulence-associated genes, whereas no CARD-annotated antimicrobial resistance genes were detected within these prophage regions. This suggests that prophages may contribute more to virulence potential and ecological adaptation than to direct antimicrobial resistance acquisition in strain A3 [56,58]. Although 13 genomic islands were also predicted, no virulence-or resistance-related genes were detected within these regions. Even so, the lack of currently annotated resistance genes within individual prophage or genomic island regions does not rule out a broader contribution of horizontal gene transfer to the evolution of this strain. In *P. mirabilis* and other Enterobacterales, insertion sequences, integrons, plasmids, and related exchange systems often work together to form and disseminate multidrug resistance regions [21,34,59]. These observations suggest that horizontal genetic exchange and genomic plasticity have likely contributed to A3’s adaptation, although the specific elements involved in resistance acquisition still require further clarification.

Li et al. [6] isolated a *P. mirabilis* strain from the feces of a diarrheic cynomolgus monkey in Yunnan Province, China. Following intraperitoneal inoculation, all challenged mice died within 24 h, while the remaining animals exhibited severe clinical abnormalities, indicating substantial pathogenicity of the isolate. Similarly, Herout et al. [47] demonstrated the pathogenic role of *P. mirabilis* in a murine model of catheter-associated urinary tract infection (CAUTI), further supporting its importance as a pathogen in urinary tract infections across multiple host species. Sun et al. [49] collected fecal swab samples from diarrheic animals on different farms in northeastern China and evaluated the pathogenicity of the recovered *P. mirabilis* isolates in mice. Within 24 h post-infection, mice challenged with both biofilm-positive and biofilm-negative isolates developed severe clinical signs, and mortality was observed in the biofilm-positive group. By 48 h, 11 mice had died, suggesting that the high pathogenicity of these isolates may be associated with the expression of multiple virulence-related genes, including *ureC*, *zapA*, *rsmA*, *hmpA*, *mrpA*, *atfA*, and *pmfA*. Hu et al. [50] isolated *P. mirabilis* strains from diarrheic dogs with bacterial enteritis between 2015 and 2017 and reported marked pathogenicity in mice, with an LD50 value of 0.57 × 10^6^ CFU/mL. In addition, Chen et al. [60] reported that multidrug-resistant *P. mirabilis* isolated from a critically endangered Malayan pangolin exhibited high virulence in a mouse infection model. In the present study, the mouse infection experiment demonstrated the substantial pathogenic potential of strain A3 under experimental conditions. All mice in the high-dose group died within 12 h following challenge, whereas only one mouse in the low-dose group died within 12 h. Histopathological examination revealed varying degrees of tissue damage in the heart, liver, spleen, lungs, and kidneys of deceased animals, and the inoculated strain was successfully re-isolated from infected organs. These findings indicate that strain A3 can cause systemic infection and multisystemic injury when administered intraperitoneally at a sufficiently high dose.

These findings alone are insufficient to establish strain A3 as the sole etiological agent responsible for the diarrheal symptoms observed in the source animal. Further studies using infection models that more closely resemble natural infection, particularly calf challenge experiments or host-relevant mucosal infection models, will be necessary to better define the contribution of bovine-derived *P. mirabilis* to enteric disease. At the same time, caution is needed when extrapolating these mouse infection results to natural infection in cattle. Intraperitoneal injection is useful for evaluating invasive and systemic pathogenicity in a controlled mouse model, but it does not reflect the natural course of *P. mirabilis* infection in cattle. Under field conditions, exposure is more likely to occur through the gastrointestinal tract, urinary tract, damaged skin, or other mucosal surfaces. Because intraperitoneal inoculation bypasses mucosal barriers, it cannot reproduce the early events of natural infection, such as colonization, local host interaction, and disease progression. In addition, mice are highly sensitive to the systemic effects of endotoxin from Gram-negative bacteria, which may partly explain the rapid deaths seen after challenge. Therefore, the observed lesions, mortality, and bacterial re-isolation from internal organs are better understood as evidence of the invasive and systemic pathogenic potential of strain A3, rather than as a direct representation of the infection process in beef cattle.

Overall, although strain A3 carries virulence-associated genes commonly found in uropathogenic *P. mirabilis*, the roles of these determinants are likely to be context-dependent. Beyond their well-described functions in urinary tract infection, factors involved in adhesion, colonization, motility, and host–microbe interactions may also contribute to bacterial persistence and mild intestinal disturbance under certain conditions. This may provide a possible explanation for the mild diarrheal signs observed in the naturally infected adult beef cattle, which is consistent with the opportunistic behavior of *P. mirabilis*. Taken together with previous reports, our findings suggest that *P. mirabilis* isolates from different hosts and ecological niches generally retain substantial pathogenic potential, although the clinical outcome may vary depending on host and environmental context.

### Limitations

Several limitations of this study should be acknowledged. First, this study was based on a single *P. mirabilis* isolate, strain A3, which was recovered from diarrheic adult beef cattle on a single farm. The number of sampled diarrheic adult beef cattle was also limited (*n* = 19). The findings of the present study, including the antimicrobial resistance profile, virulence gene repertoire, genomic characteristics, and pathogenicity in mice, should be interpreted as strain-specific observations for A3 rather than representative features of *P. mirabilis* as a species or of bovine-derived isolates as a whole. Although comparative genomic analysis showed high ANI similarity between A3 and other bovine-derived strains (NY1, NY2, and NY3), ranging from 99.06% to 99.32%, this genomic relatedness does not justify extrapolating the phenotypic or pathogenic characteristics of A3 to the broader population of bovine-associated *P. mirabilis*. The genetic diversity, geographical variation, host-associated differences, and farm management factors among bovine-derived *P. mirabilis* isolates remain to be clarified in future studies with larger sample sizes. Second, the animal infection experiment was performed in mice using intraperitoneal inoculation. This design allowed an initial assessment of systemic pathogenic potential under controlled conditions, but it does not reproduce the natural route of infection, host physiology, or disease progression in cattle. Under field conditions, *P. mirabilis* is more likely to enter the bovine host through the gastrointestinal tract, urinary tract, wounds, or other mucosal surfaces. The in vivo findings should therefore be interpreted as evidence of invasive pathogenic capacity in an experimental model rather than as a direct representation of the natural infection process or clinical outcome in beef cattle.

Despite these limitations, this study provides an integrated phenotypic, genomic, and pathogenicity-based characterization of a bovine-derived *P. mirabilis* isolate from Anhui Province, China. These findings provide strain-level evidence and a valuable reference for future surveillance and functional studies. Further investigations involving larger numbers of bovine-derived *P. mirabilis* isolates from different farms, regions, and production systems, together with more physiologically relevant infection models, are needed to better define the epidemiological significance and potential role of bovine-associated *P. mirabilis* in enteric disease.

## 5. Conclusions

A bovine-derived *P. mirabilis* strain (A3) was isolated from a diarrheic beef cow and comprehensively characterized using phenotypic assays, PCR screening, whole-genome sequencing, comparative genomics, and mouse infection experiments. Strain A3 exhibited a multidrug-resistant phenotype and carried multiple resistance-associated genes, while genome analysis identified virulence-related determinants linked to adhesion, motility, toxin-associated activity, iron acquisition, and host interaction. Comparative genomic analysis confirmed the taxonomic identity of strain A3 (ANI > 95% versus reference *P. mirabilis* genomes), showed close relatedness to other bovine isolates, and indicated an open pangenome structure, supporting substantial genomic plasticity at the species level. In vivo challenge demonstrated dose-dependent pathogenicity and dose-dependent lethality, with successful re-isolation of the inoculated strain from major organs. Collectively, these findings indicate that bovine-associated *P. mirabilis* A3 combines multidrug resistance with opportunistic pathogenic potential. Although the original host showed only mild diarrhea, and causality cannot be confirmed from a single isolate, the virulence repertoire of A3 provides a plausible basis for mucosal colonization, intestinal disturbance, and systemic pathogenicity under experimental conditions. This work expands current knowledge of *P. mirabilis* in beef cattle and provides genomic and experimental evidence for surveillance, risk assessment, and prevention strategies in livestock production systems. However, because this study was based on a single bovine-derived isolate from one farm, these findings should be interpreted as strain-level evidence and should not be generalized to all bovine-associated *P. mirabilis* isolates.

## Figures and Tables

**Table 1:** PCR Primers for drug resistance genes and virulence genes of *P. mirabilis* strain A3.

VFs and ARGs	Type	Genes	Primer Sequence (5′→3′)	Target Fragment Size (bp)	Annealing Temperature/°C
Virulence genes	Metabolic enzyme	*ureC*	F: GTTATTCGTGATGGTATGGG R: ATAAAGGTGGTTACGCCAGA	373	52
	Adhesion (fimbrial)	*mrpA*	F: ATTTCAGGAAACAAAAGATG R: TTCTTACTGATAAGACATTG	184	40
		*ucaA*	F: GTAAAGTTGTTGCGCAAAC R: TTGAGCCACTGTGGATACA	246	50
		*pmfA*	F: GGATCATCTATAATGAAACTG R: CTGATAATCAACTTGGAAGTT	512	52
		*atfA*	F: CATAATTTCTAGACCTGCCCTAGCA R: CTGCTTGGATCCGTAATTTTTAACG	332	50
	Virulence regulation	*rsbA*	F: TCGATTTCAGTGTTTGGCCAT R: CCGAGCTTCATCATGGCTG	655	56
		*atfC*	F: AGAAAGGGATCCTACAAATTAA R: TATAGCATGCATTTAAATTGCC	688	50
Drug resistance genes	β-lactams	*CTX-M*	F: TTTGCGATGTGCAGTACCAGTAA R: TTTGCGATGTGCAGTACCAGTAA	543	55
		*TEM*	F: TACGATACGGGAGGGCTTAC R: TTCCTGTTTTTGCTCACCCA	716	56
		*SHV*	F: TCAGCGAAAAACACCTTG R: TCCCGCAGATAAATCACCA	471	55
	Quinolones	*qnrA*	F: ATTTCTCACGCCAGGATTTG R: GATCGGCAAAGGTTAGGTCA	516	52
		*qnrB*	F: GATCGTGAAAGCCAGAAAGG R: ATGAGCAACGATGCCTGGTA	476	63
		*qnrS*	F: GCAAGTTCATTGAACAGGGT R: TCTAAACCGTCGAGTTCGGCG	428	55
	Aminoglycosides	*aac-Ib*	F: TTGCGATGCTCTATGAGTGGCTA R: CTCGAATGCCTGGCGTGTTT	482	53

**Table 2:** Phenotypic and biochemical characteristics of *P. mirabilis* strain A3.

Test	Result	Test	Result
Semi-solid agar	+	V P	+
Ornithine decarboxylase broth	+	Phenylalanine	+
Lysine decarboxylase broth	−	Mannitol	−
Amino acid decarboxylase control	−	Inositol	−
Simmons citrate	+	Sorbitol	−
Hydrogen sulfide (H_2_S)	+	Melibiose	−
Urease	+	Adonitol	−
Peptone water (tryptophan broth)	−	Raffinose	−
M R	+	/	/

Note: The phenotypic and biochemical characteristics were determined for the single bovine-derived isolate A3. “+” indicates a positive result, and “−” indicates a negative result.

**Table 3:** Antimicrobial susceptibility test results of the *P. mirabilis* strain A3.

Antimicrobial Class	Antimicrobial Agent	Diameter of Inhibition Zone (mm)	Interpretation	Interpretive Criteria (mm) [27]		
				R	I	S
β-lactams	Ceftriaxone	35	S	≤19	20~22	≥23
	Cefazolin	10	R	≤14	15~17	≥18
	Cefoperazone	27	S	≤15	16~20	≥21
	Cefuroxime	14	R	≤14	15~17	≥18
	Ceftazidime	14	R	≤17	18~20	≥21
	Cefalexin	10	R	≤14	15~17	≥18
	Ampicillin	0	R	≤13	14~16	≥17
	Piperacillin	23	S	≤17	18~20	≥21
	Cefradine	0	R	≤14	15–17	≥18
	Carbenicillin	18	R	≤19	20–22	≥23
	Imipenem	0	R	≤19	20–22	≥23
Aminoglycosides	Amikacin	20	S	≤14	15~16	≥17
	Kanamycin	29	S	≤13	14~17	≥18
	Gentamicin	25	S	≤12	13~14	≥15
	Neomycin	12	I	≤11	12~14	≥15
Tetracyclines	Doxycycline	13	S	≤9	10~12	≥13
	Minocycline	15	I	≤12	13~15	≥16
	Tetracycline	9	R	≤11	12~14	≥15
Quinolones	Ofloxacin	29	S	≤12	13~15	≥16
	Ciprofloxacin	25	S	≤15	16~20	≥21
	Norfloxacin	34	S	≤12	13~16	≥17
Macrolides	Erythromycin	14	I	≤11	12~17	≥18
	Midecamycin	0	R	≤13	14~17	≥18
	Azithromycin	20	S	≤14	15~17	≥18
Amide alcohols	Chloramphenicol	20	S	≤12	13~17	≥18
Sulfonamides	Trimethoprim-sulfamethoxazole	22	S	≤10	11~15	≥16

Note: R, resistant; I, intermediate; S, susceptible. Susceptibility testing was performed for the single bovine-derived isolate A3, with technical replicates conducted according to the testing protocol.

**Table 4:** Genome component analysis of *P. mirabilis* strain A3.

Sample ID	A3
Number of assembled sequences	76
Total assembly length	3,965,086 bp
Number of ambiguous bases	1000
Proportion of ambiguous bases	0.00025
GC content %	38.37%
Maximum sequence length	455,889
Minimum sequence length	501
Sequences > 1 kb	63

Note: Genome statistics were derived from whole-genome sequencing and assembly of the bovine-derived isolate A3 analyzed in this study (biological replicate, *n* = 1 isolate).

**Table 5:** Genomic plasticity elements of *P. mirabilis* strain A3.

Element Category	Specific Type/Family	Count	Total Length (bp)	Major Scaffold Distributions	Remarks
Insertion sequences (IS)	IS200/IS605	4	2211	10, 15, 3, 6	Contains complete transposase, potentially active
	IS21	3	10,271	11, 3, 7	Possess inverted repeats
	IS3	2	1670	2, 5	/
	IS481	1	406	14	/
	ISNCY	1	1143	28	High density on scaffold28
Prophage	Complete/partial	12	~567 kb ^1^	1, 2, 3, 4, 5, 6, 7, 8, 11, 12, 14	All possess attL/attR, potentially integrative
Virulence genes carried by the prophage	VFDB annotated	22	/	pp6, pp10, pp15, pp18, pp19	pp15 carries the most (>10)
Genomic islands	Predicted regions	13	~102 kb ^2^	3, 4, 5, 6, 7, 11, 12, 17, 28, 31	No virulence/resistance genes found
CRISPR-Cas	CRISPR loci	5	1115	2, 3, 5, 8, 18	The largest locus has 7 spacers
Transposon homologous fragments	ISC1316	4	1341	6, 10, 15	/
/	LINE element	1	429	5	/

Notes: ^1^ Estimated total length of all prophages; ^2^ Total length of genomic islands estimated from their start and end positions. Identification of insertion sequences, prophage regions, genomic islands, CRISPR loci, and related mobile genetic elements was performed based on the genome sequence of the bovine-derived isolate A3 analyzed in this study (biological replicate, *n* = 1 isolate).

**Table 6:** Analysis of resistance genes of *P. mirabilis* strain A3.

Antibiotic Class	Number of Genes	Resistance Mechanism	Resistance Genes
β-lactams	22	Antibiotic efflux pump, target modification	*acrB*, *MexAB*, *MexB*, *golS*, *marA*, *ramA*, *PBP3 mutant*
Fluoroquinolones	18	DNA gyrase/topoisomerase mutation, efflux pump	*GyrB mutant*, *parE mutant*, *parC mutant*, *emrR*, *mdtH*, *MexG*
Tetracyclines	15	Ribosomal protection, efflux pump	*tet(J)*, *tetR*, *rpsJ*, *acrB*, *MexAB*
Polypeptide antibiotics	14	Target modification, efflux pump	*arnA*, *ugd*, *PmrF*, *rosB*, *LpxA*, *LpxC*, *OmpA*
Macrolides	12	Efflux pump, ribosomal methylation	*macB*, *abeS*, *MexAB*, *KpnE*, *KpnF*
Aminoglycosides	9	Efflux pump, target modification	*acrB*, *MexAB*, *baeR*, *baeS*, *cpxA*, *rpsL mutant*
Phenicols	8	Antibiotic inactivation, efflux pump	*catB9*, *catA4*, *acrB*, *MexAB*
Rifamycins	7	RNA polymerase mutation, efflux pump	*RpoB mutant*, *acrB*, *ramA*
Carbapenems	6	Efflux pump, porin loss	*MexAB*, *MexB*, *marA*, *ramA*, *LptD*
Fosfomycins	5	Target modification	*MurA mutant*, *GlpT mutant*, *UhpA mutant*, *PtsI mutant*
Glycopeptides	4	Target modification	*RpoC mutant*, *rpld*, *mlaF*, *mlaD*
Disinfectants/antiseptics	8	Efflux pump, target modification	*acrB*, *MexG*, *leuO*, *fabI mutant*

Notes: Resistance-associated genes were annotated based on whole-genome analysis of the bovine-derived isolate A3 analyzed in this study (biological replicate, *n* = 1 isolate).

**Table 7:** Analysis of virulence genes of *P. mirabilis* strain A3.

VF Category	Number of Genes	Subcategory/System	Representative Genes/Gene Clusters
Adhesion	33	MR/P fimbriae	*mrpA*, *mrpB*, *mrpC*, *mrpD*, *mrpH*, *mrpI*, *mrpJ*, etc.
		PMF fimbriae	*PMI_RS09265*, *PMI_RS09270*, *PMI_RS09275*, *PMI_RS09280*, *PMI_RS09285*
		UCA fimbriae	*ucaA*, *PMI_RS02645*, *PMI_RS02640*, *PMI_RS02635*, *PMI_RS02630*
		Other adhesins	*fimA*, *fimC*, *fimD*, *fimH*, *ompA*, *dnaK*, *IlpA*, *lmb*, etc.
Motility	32	Flagellar structural components	*flaA(FliC)*, *flgB/C/F/G/H/I*, *fliD*, etc.
		Flagellar assembly and export	*flhA/B*, *fliI/J/P/Q/R*, etc.
		Flagellar motor and switch	*motA/B*, *fliG/M/N*
		Flagellar gene regulation	*fliA(σ28)*, *fliZ*, *flhC*, *flhD*
		Chemotaxis system	*cheA/B/R/W/Y/Z*, *cheD*
Nutritional/metabolic factors	29	Heme uptake (HmuR2)	*PMI_RS06900*, *PMI_RS06905*, *PMI_RS06910*, *PMI_RS06915*, *PMI_RS06920*
		Other iron transport and regulation	*chuA*, *hitC*, *fbpB*, *feoB*, *fur (regulator)*
		Bacteriocin	*(Proteobactin)Gene cluster PMI_RS01125-PMI_RS01160*, etc.
		Basal metabolic adaptation	*hem series*, *narG*, *narH*
Immune modulation	27	LPS/LOS biosynthesis	*lpxB/C*, *rfaE/F*, *gmhA*, *galU/E*, *gtrB*
Effector delivery system	11	Type VI secretion system (T6SS)	*hcp*, *vgrG3*, *clpV*
		Hemolysin system (HpmAB)	*hpmA*, *hpmB*
		Autotransporter adhesin/toxin	*aipA*, *pta*
		Metalloprotease	*zapA(PMI_RS01355)*
Stress survival	5	Oxidative stress resistance	*sodB*, *katA*
		Acid/base adaptation	*ureB*, *ureG (urease-related)*
Regulation	5	Global/two-component regulation	*rpoS(σS)*, *fur*, *csrA*, *rcsB*, *bvgA*
Antimicrobial activity/Competitive advantage	2	/	*acrB(efflux pump)*
Biofilm	2	/	*algU*, *mucC*
Exotoxin	2	/	*hpmA*, *zapA*
Others	1	/	*Unclassified*

Notes: Virulence-associated genes were annotated based on whole-genome analysis of the bovine-derived isolate A3 analyzed in this study (biological replicate, *n* = 1 isolate).

## Data Availability

The original contributions presented in this study are included in the article. Further inquiries can be directed to the corresponding author.
